# Comparative proteome analysis of *Milnesium tardigradum* in early embryonic state *versus* adults in active and anhydrobiotic state

**DOI:** 10.1371/journal.pone.0045682

**Published:** 2012-09-27

**Authors:** Elham Schokraie, Uwe Warnken, Agnes Hotz-Wagenblatt, Markus A. Grohme, Steffen Hengherr, Frank Förster, Ralph O. Schill, Marcus Frohme, Thomas Dandekar, Martina Schnölzer

**Affiliations:** 1 Functional Proteome Analysis, German Cancer Research Center (DKFZ), Heidelberg, Germany; 2 Department of Molecular Biology and Functional Genomics, University of Applied Sciences Wildau, Wildau, Germany; 3 Department of Zoology, University of Stuttgart, Stuttgart, Germany; 4 Department of Bioinformatics, University of Würzburg, Würzburg, Germany; Berlin Institute of Technology, Germany

## Abstract

Tardigrades have fascinated researchers for more than 300 years because of their extraordinary capability to undergo cryptobiosis and survive extreme environmental conditions. However, the survival mechanisms of tardigrades are still poorly understood mainly due to the absence of detailed knowledge about the proteome and genome of these organisms. Our study was intended to provide a basis for the functional characterization of expressed proteins in different states of tardigrades. High-throughput, high-accuracy proteomics in combination with a newly developed tardigrade specific protein database resulted in the identification of more than 3000 proteins in three different states: early embryonic state and adult animals in active and anhydrobiotic state. This comprehensive proteome resource includes protein families such as chaperones, antioxidants, ribosomal proteins, cytoskeletal proteins, transporters, protein channels, nutrient reservoirs, and developmental proteins. A comparative analysis of protein families in the different states was performed by calculating the exponentially modified protein abundance index which classifies proteins in major and minor components. This is the first step to analyzing the proteins involved in early embryonic development, and furthermore proteins which might play an important role in the transition into the anhydrobiotic state.

## Introduction

Tardigrades are small invertebrates with a body length of 0.1–1.0 mm. *Milnesium tardigradum* Doyère (1840) belongs to the species of carnivorous tardigrades and is analyzed regarding different aspects of its life history [1], [2]. Tardigrades have been in focus in the last decades because of their amazing capability to undergo anhydrobiosis and survive physical extremes including high and subzero temperatures [3], [4], [5], [6], high pressure [5], [7] and extreme levels of ionizing radiation [8], [9], [10]. There are two known strategies to cope with water deficiency: “desiccation-avoidance strategy” and “desiccation-tolerance strategy” [11]. The term “desiccation-avoidance strategy” describes physiological and morphological adaptations to reduce water loss. For example the African lungfish build a waterproof cocoon to prevent the over-dehydration [11]. “Desiccation-tolerance strategy” is used for withstanding the dehydrated state. The best example is anhydrobiosis, when the metabolic activity is reversibly at a standstill. Thereby, tardigrades contract their legs and build the so-called tun [12], in which they are resistance to extreme environmental conditions.

Even though detailed aspects of the life cycle of tardigrades are already described, there remains a notable absence of detailed knowledge concerning the proteome and genome of these animals, which provides the basis for further investigations including developmental analysis and also characterizing the molecular mechanisms of the protections and survival mechanisms in tardigrades during anhydrobiosis. With our investigation we intended to fill this gap by performing shotgun proteomics on tardigrades using 1D-SDS-PAGE and high sensitivity nanoLC-ESI-MS/MS on an LTQ-Orbitrap mass spectrometer.

Up to date there are only few published transcriptomic [13], [14] and proteomic [15], [16] studies available, which were carried out using EST sequences generated by Sanger sequencing from *M. tardigradum*. Using a newly established EST database based on 454 sequencing, we present in this study a comprehensive comparative analysis of the proteome of tardigrades in three different states: early embryonic state (EES), adult tardigrades in active (AS) and anhydrobiotic (tun) state (TS). More than 3000 proteins were identified with high sequence coverage. This comprehensive proteome resource includes different protein families such as chaperones, antioxidants, ribosomal proteins, cytoskeletal proteins, transporters, protein channels, nutrient reservoirs, and developmental proteins. In addition proteins such as Late Embryogenesis Abundant protein (LEA), which were previously identified by homology search against the NCBInr database [15] are now characterized by MS/MS analysis using the *M. tardigradum* database for the first time.

Our study presents not only a milestone in analyzing the proteome of tardigrades, but also a comparative analysis of different states of tardigrades using a label-free semi-quantification method. All proteins were quantified by calculating their exponentially modified Protein Abundance Index (emPAI), which allows the classification of proteins in major and minor components and thereby a semi-quantitative analysis of differentially expressed proteins in different states. Applying this method, we firstly compared the proteome of tardigrades in early embryonic state *versus* adult tardigrades (in both active and tun state) and secondly adult tardigrades in active state *versus* tun state.

## Results

### Identification and Classification of Proteins Expressed in *M. tardigradum*

**Figure 1:**
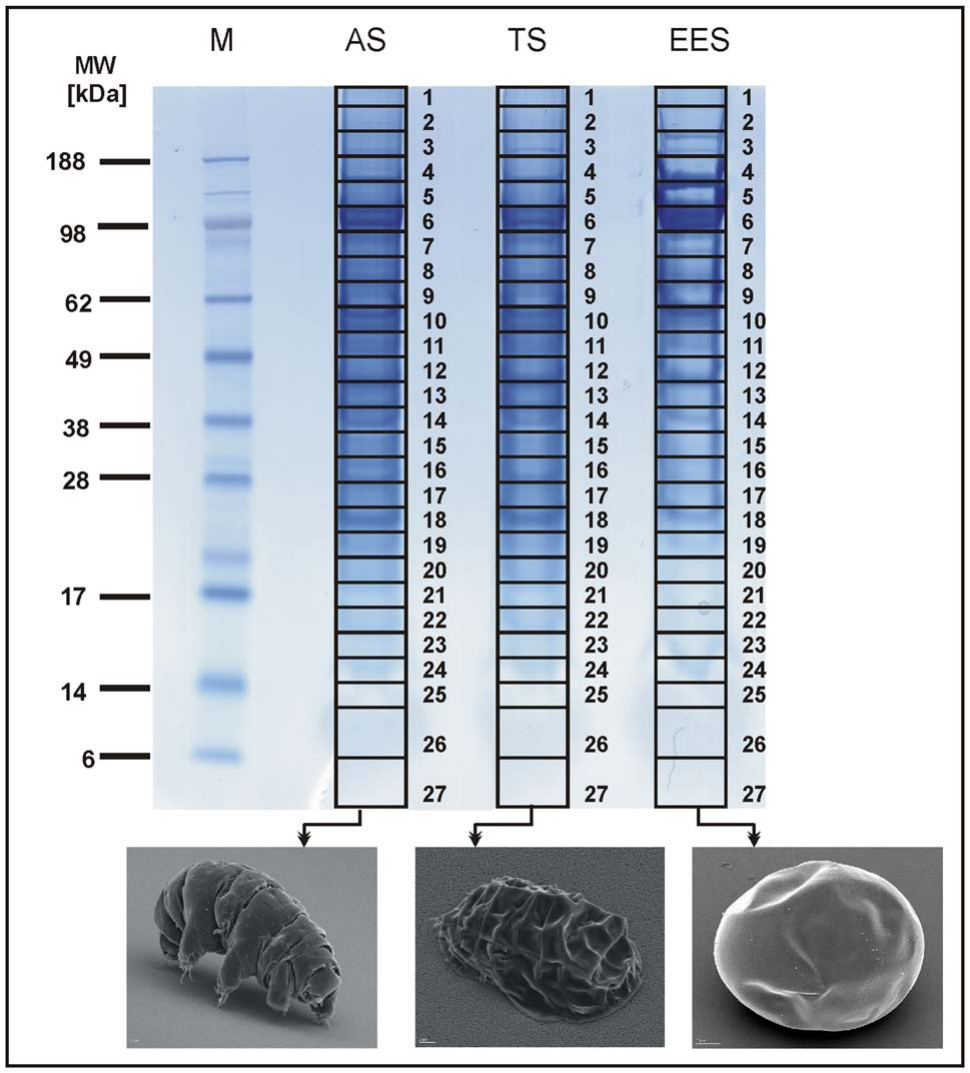
Separation of protein lysates of tardigrades in three different states by one-dimensional polyacrylamide gel electrophoresis. Lane 1: Rainbow molecular weight marker. Lane 2: Protein extract of adult tardigrades in active state (AS). Lane 3: Protein extract of adult tardigrades in tun state (TS). Lane 4: Protein extract of tardigrades in early embryonic state (EES). Bottom. SEM-images of *M. tardigradum* in the corresponding states.

One dimensional gel electrophoresis in combination with high sensitive nanoLC-ESI-MS/MS allowed us the identification of proteins on a large scale. We investigated the proteome of *M. tardigradum* in early embryonic state (EES) and of adult animals in active (AS) and tun state (TS) (Figure 1). The analysis yielded 1982 proteins in EES, 2345 proteins in AS and 2281 proteins in TS. The complete results of database searches and protein identifications for each state including decoy analysis are provided in Tables S1 (EES), S2 (AS) and S3 (TS). Identifications based on one peptide were allowed only in cases we found the same protein in different gel slices. By setting the search parameters as such that they refer to a match probability of p<0.01, we minimized the false discovery rate (FDR) to values below 5%. Only the FDRs in gel slices in the low molecular weight range (e.g. slice 26 and 27) were higher than 5%. Since proteins identified in these slices were mostly one peptide identifications, they were excluded from further analyses. Database search of the MS/MS spectra resulted in proteins that could be separated into two groups: identified proteins with annotation (annotated by Blast search against SwissProt and NCBInr databases) and those without annotation. Proteins with annotation were classified into different functional groups defined by gene ontology using Blast2GO program. A summary of all identified proteins and their classification in selected protein families and functional groups is given in Table S4. A broad range of diverse protein families including chaperones, antioxidants, ribosomal proteins, cytoskeletal and motor proteins, transporters, protein channels, nutrient reservoirs, and developmental proteins are present in the results. Identified proteins, which could not be annotated using homology search against the SwissProt and NCBInr database were analyzed for specific protein domains using DomainSweep. A total of 1135 contigs without annotation were identified including one-peptide identifications. The DomainSweep analysis resulted in 129 proteins, which showed significant protein domains. For another 455 proteins we found putative protein domains. For the remaining 551 contigs we could not receive any information. The result of DomainSweep analysis for not annotated proteins identified with more than one peptide is available in Table S4.

### Determination of Major Components in Early Embryonic State and of Adult Animals in Active and Tun State

The comparative analysis of tardigrades in different states was performed using a label-free technique based on emPAI. The emPAI (exponentially modified Protein Abundance Index) is defined as the ratio of the number of identified tryptic peptides to the number of theoretically observable tryptic peptides for each protein [17]. In our study the emPAI (included in Tables S1, S2 and S3) was only used to give an approximate estimate of relative protein concentration to grouping the proteins into minor and major components for each state. Thus, our data provide an overview of protein classes, which are highly abundant in each state.

Selected proteins associated with diverse processes such as response to stimulus, protection and development were compared based on their emPAI. Data are summarized in Table 1.

**Table 1:** Semi-quantitative analysis of selected proteins associated with diverse processes such as response to stimulus, protection and development.

GO term (GO category, GO level)	Protein annotation	Contig no.	Accession no.	empAI		
				EES	AS	TS
**Response to stress (BP, 3)**	similar to Heat shock protein HSP 90-alpha (P07900)|Evalue: 6e-93	contig26899:1:722:2	EZ763093	/	0,26	0,26
	similar to Heat shock protein 81-2 (Q69QQ6)|Evalue: 8e-163	contig20254:1:1418:3	EZ761693	/	/	0,32
	similar to Endoplasmin (P08113)|Evalue: 2e-101	contig02760:1:1133:2	EZ759412	0,59	2,67	1,37
	similar to Heat shock cognate 70 kDa protein 2 (P27322)|Evalue: 7e-146	contig24452:1:1324:1	EZ762552	/	0,29	0,63
	similar to Heat shock cognate 71 kDa protein (P19120)|Evalue: 2e-47	contig12271:1:402:2	EZ760741	7,85	10,32	5,73
	similar to Heat shock 70 kDa protein cognate 4 (Q9U639)|Evalue: 9e-73	contig07914:1:483:1	EZ760246	2,27	5,82	4,42
	similar to Heat shock 70 kDa protein 4 (P34932)|Evalue: 1e-15	contig06011:1:452:3	EZ759976	3,06	4,03	3,84
	similar to Heat shock 70 kDa protein 4 (P11145)|Evalue: 2e-90	contig24686:114:732:3	EZ762618	0,99	1,5	2
	similar to Heat shock 70 kDa protein A (P09446)|Evalue: 2e-54	contig15471:1:392:1	EZ760913	/	1,15	0,91
	similar to Protein lethal(2)essential for life (P82147)|Evalue: 1e-12	contig01972:1:390:1	EZ759252	1,02	2,53	3,5
	similar to 10 kDa heat shock protein, mitochondrial (Q5DC69)|Evalue: 1e-34	contig08799:2167:2550:3	EZ760357	2,53	/	/
	similar to Small heat shock protein C4 (Q4UJB1)|Evalue: 1e-17	contig04304:1:362:3	EZ759702	0,56	/	/
	similar to Major egg antigen (P12812)|Evalue: 1e-05	contig20502:1:416:2	EZ761736	0,45	/	/
	similar to Stress-induced-phosphoprotein 1 (Q4R8N7)|Evalue: 7e-84	contig16533:1:782:3	EZ760961	0,89	1,1	1,34
	similar to MAP kinase-activated protein kinase 3 (Q16644)|Evalue: 7e-136	contig18632:105:1412:3	EZ761329	/	0,21	0,14
	similar to Sec1 family domain-containing protein 1 (Q62991)|Evalue: 8e-178	contig27900:171:2096:3	EZ763258	/	0,09	0,19
	similar to Mitogen-activated protein kinase 14A (O62618)|Evalue: 2e-54	contig20576:1:610:2	EZ761746	0,31	/	/
	similar to 5∼-AMP-activated protein kinase subunit beta-2 (O43741)|Evalue: 3e-69	contig07985:2341:3165:3	EZ760258	0,51	0,51	0,51
	similar to STE20/SPS1-related proline-alanine-rich protein kinase (O88506)|Evalue: 7e-152	contig18837:256:1929:1	EZ761386	0,11	/	0,11
	similar to Citrate synthase, mitochondrial (Q28DK1)|Evalue: 0.0	contig08799:1:1630:2	EZ760357	2,39	7,86	3,91
	similar to Eukaryotic translation initiation factor 2 subunit 1 (Q5R493)|Evalue: 4e-88	contig25843:96:1024:3	EZ762896	2,76	3,42	3,44
	similar to Translation initiation factor eIF-2B subunit alpha (Q99LC8)|Evalue: 2e-14	contig27961:1:347:1	EZ763267	0,86	/	1,02
	similar to Translation initiation factor eIF-2B subunit gamma (P70541)|Evalue: 3e-50	contig02684:38:859:2	EZ759398	/	/	0,22
	similar to Translation initiation factor eIF-2B subunit epsilon (Q8CHW4)|Evalue: 1e-61	contig08308:1:904:1	EZ760299	/	/	0,2
	similar to Translation initiation factor eIF-2B subunit alpha (Q14232)|Evalue: 3e-59	contig26081:1:614:2	EZ762945	/	0,74	/
	similar to Translation initiation factor eIF-2B subunit delta (P41111)|Evalue: 2e-102	contig25423:124:1422:1	EZ762793	0,14	0,22	0,31
	similar to Protein DJ-1 (Q5E946)|Evalue: 8e-08	contig27778:718:913:2	EZ763236	0,96	2,27	2,27
	**Total emPAI**			**28,25**	**44,99**	**36,64**
**Response to hypoxia (BP, 3)**	similar to 60 kDa heat shock protein, mitochondrial (P63038)|Evalue: 0.0	contig07413:1:1541:1	EZ760182	10,73	13,95	12,12
	similar to Endoplasmin (Q95M18)|Evalue: 1e-143	contig00854:1:1427:3	EZ759053	1,65	3,04	2,56
	similar to Glycogen phosphorylase, liver form (Q0VCM4)|Evalue: 1e-37	contig11894:71:359:2	EZ760699	/	1,19	0,99
	similar to Niemann-Pick C1 protein (P56941)|Evalue: 7e-175	contig19457:1:1683:1	EZ761529	/	0,1	0,1
	similar to Lon protease homolog, mitochondrial (P36776)|Evalue: 1e-159	contig03034:1:1882:2	EZ759466	/	0,09	/
	similar to Probable V-type proton ATPase 116 kDa subunit a (P30628)|Evalue: 2e-28	contig08886:1:238:1	EZ760368	0,74	/	0,74
	similar to Hypoxia up-regulated protein 1 (Q9Y4L1)|Evalue: 2e-24	contig23865:1:793:3	EZ762386	/	/	0,24
	**Total emPAI**			**13,12**	**18,37**	**16,75**
**Response to heat (BP, 3)**	similar to Heat shock cognate 71 kDa protein (Q90473)|Evalue: 0.0	contig07915:1:1267:2	EZ760247	30,43	32,48	21,57
	similar to Heat shock protein HSP 90-alpha (Q76LV2)|Evalue: 0.0	contig25094:2604:4781:3	EZ762716	21,33	19,82	13,85
	similar to Fructose-bisphosphate aldolase, muscle type (P53445)|Evalue: 3e-36	contig25519:1:245:2	EZ762820	2,52	19,21	24,28
	similar to Fructose-bisphosphate aldolase A (P05065)|Evalue: 6e-54	contig05470:1:464:3	EZ759889	3,69	16,75	14,34
	similar to 60 kDa heat shock protein, mitochondrial (P63038)|Evalue: 0.0	contig07413:1:1541:1	EZ760182	10,73	13,95	12,12
	similar to Chaperone protein dnaK (A6T4F4)|Evalue: 0.0	contig03976:126:2186:3	EZ759653	4,36	8,48	8,05
	similar to Superoxide dismutase [Cu-Zn] (Q8HXQ3)|Evalue: 5e-52	contig17128:1:666:1	EZ761032	10,94	7,3	5,72
	similar to Superoxide dismutase [Cu-Zn] (O73872)|Evalue: 7e-25	contig10844:1:307:2	EZ760584	29,04	6,87	15,04
	similar to Pyrroline-5-carboxylate reductase (P54904)|Evalue: 5e-61	contig23741:298:1137:1	EZ762351	0,53	1,13	0,53
	similar to Translation initiation factor eIF-2B subunit alpha (Q14232)|Evalue: 3e-59	contig26081:1:614:2	EZ762945	/	0,74	/
	similar to Heat shock protein beta-1 (P42930)|Evalue: 5e-12	contig11265:1:246:1	EZ760631	/	0,74	0,74
	similar to DnaJ homolog subfamily B member 1 (P25685)|Evalue: 2e-62	contig01190:1:651:1	EZ759115	/	0,48	0,3
	similar to Translation initiation factor eIF-2B subunit beta (Q90511)|Evalue: 6e-96	contig23557:134:1258:2	EZ762299	0,36	0,26	0,16
	similar to Glutamate–cysteine ligase catalytic subunit (P48506)|Evalue: 7e-128	contig24640:1:1499:3	EZ762603	/	0,25	/
	similar to Translation initiation factor eIF-2B subunit delta (P41111)|Evalue: 2e-102	contig25423:124:1422:1	EZ762793	0,14	0,22	0,31
	similar to Glutamate–cysteine ligase catalytic subunit (P48506)|Evalue: 6e-120	contig27962:63:1574:3	EZ763268	/	0,12	0,39
	similar to Glutamate–cysteine ligase catalytic subunit (P97494)|Evalue: 0.0	contig05957:1:1687:2	EZ759966	/	/	0,41
	similar to Translation initiation factor eIF-2B subunit gamma (P70541)|Evalue: 3e-50	contig02684:38:859:2	EZ759398	/	/	0,22
	similar to Translation initiation factor eIF-2B subunit epsilon (Q8CHW4)|Evalue: 1e-61	contig08308:1:904:1	EZ760299	/	/	0,2
	similar to Translation initiation factor eIF-2B subunit alpha (Q99LC8)|Evalue: 2e-14	contig27961:1:347:1	EZ763267	0,86	/	1,02
	**Total emPAI**			**114,93**	**128,8**	**119,25**
**Response to oxidative stress (BP, 3)**	similar to Catalase HPII (P21179)|Evalue: 0.0	contig01298:1:2065:3	EZ759139	7,24	13,23	11,24
	similar to Isocitrate dehydrogenase [NADP] cytoplasmic (Q9XSG3)|Evalue: 8e-107	contig25483:1682:2388:3	EZ762808	3,77	11,5	4,62
	similar to Peroxiredoxin-2 (Q8K3U7)|Evalue: 7e-29	contig21646:1:212:3	EZ761908	3,63	7,06	3,92
	similar to Glutamate–cysteine ligase regulatory subunit (P48508)|Evalue: 9e-40	contig02105:90:857:3	EZ759284	2,14	4,7	5,66
	similar to Peroxidasin (A4IGL7)|Evalue: 7e-54	contig22552:1:2159:2	EZ762049	/	3,79	2,75
	similar to Peroxiredoxin-2 (Q2PFZ3)|Evalue: 2e-33	contig24044:157:536:1	EZ762425	4,02	3,19	4,62
	similar to Protein DJ-1 (Q5E946)|Evalue: 8e-08	contig27778:718:913:2	EZ763236	0,96	2,27	2,27
	similar to Peroxiredoxin-5, mitochondrial (P30044)|Evalue: 2e-48	contig20474:30:605:3	EZ761733	0,9	1,15	0,72
	similar to Chloride intracellular channel exc-4 (Q8WQA4)|Evalue: 2e-50	contig26932:43:801:1	EZ763098	1,08	1,11	0,81
	similar to Thioredoxin reductase 1, cytoplasmic (Q16881)|Evalue: 1e-165	contig28039:246:1925:3	EZ763281	0,34	1,08	0,73
	similar to Peroxiredoxin-2 (Q2PFZ3)|Evalue: 5e-75	contig17026:72:905:3	EZ761016	0,59	1,02	1,2
	similar to Peroxidasin (Q9VZZ4)|Evalue: 6e-41	contig11268:1:905:3	EZ760633	0,3	0,85	0,45
	similar to Proteasome subunit beta type-5 (P28075)|Evalue: 1e-41	contig25696:1:661:3	EZ762858	0,95	0,75	0,96
	similar to Dual specificity mitogen-activated protein kinase kinase 2 (P36506)|Evalue: 7e-17	contig05837:177:528:3	EZ759953	0,6	0,6	/
	similar to Peroxidasin homolog (Q3UQ28)|Evalue: 6e-59	contig07623:1:1128:1	EZ760209	/	0,56	0,56
	similar to Chorion peroxidase (P82600)|Evalue: 4e-35	contig25378:1:753:2	EZ762777	/	0,37	0,12
	similar to Dual specificity mitogen-activated protein kinase kinase 1 (Q91447)|Evalue: 9e-94	contig20126:1:839:3	EZ761665	/	0,32	0,22
	similar to Glutamate–cysteine ligase catalytic subunit (P97494)|Evalue: 2e-33	contig21482:1:542:2	EZ761881	/	0,32	/
	similar to Glutamate–cysteine ligase catalytic subunit (P48506)|Evalue: 7e-128	contig24640:1:1499:3	EZ762603	/	0,25	/
	similar to Torsin-like protein (Q95NU5)|Evalue: 1e-63	contig17495:1:1150:2	EZ761080	0,24	0,24	0,16
	similar to Glutamate–cysteine ligase catalytic subunit (P48506)|Evalue: 6e-120	contig27962:63:1574:3	EZ763268	/	0,12	0,39
	similar to Nicotinate phosphoribosyltransferase (Q9VQX4)|Evalue: 0.0	contig00602:234:2048:3	EZ759010	0,21	0,1	0,21
	similar to Chorion peroxidase (Q9VEG6)|Evalue: 6e-75	contig04779:1:1782:1	EZ759779	/	0,1	0,1
	similar to Inositol 1,4,5-trisphosphate receptor type 1 (Q14643)|Evalue: 1e-84	contig04295:229:1159:1	EZ759699	0,18	0,09	0
	similar to Lon protease homolog, mitochondrial (P36776)|Evalue: 1e-159	contig03034:1:1882:2	EZ759466	/	0,09	/
	similar to Multidrug resistance-associated protein 1 (Q8HXQ5)|Evalue: 2e-80	contig24886:88:1362:1	EZ762662	/	/	0,14
	similar to 40S ribosomal protein S14 (P19951)|Evalue: 1e-51	contig26340:1:429:1	EZ762999	0,74	/	/
	similar to Glutamate–cysteine ligase catalytic subunit (P97494)|Evalue: 0.0	contig05957:1:1687:2	EZ759966	/	/	0,41
	similar to Peroxiredoxin-5, mitochondrial (Q9GLW9)|Evalue: 2e-44	contig18110:116:595:2	EZ761225	0,42	/	/
	**Total emPAI**			**28,31**	**54,86**	**42,26**
**Transmembrane transporter activity (MF, 3)**						
	**glucose import**					
	similar to Solute carrier family 2, facilitated glucose transporter member 1 (P27674)|Evalue: 5e-108	contig02938:228:1706:3	EZ759449	0,42	0,54	0,42
	**anion transport**					
	similar to Mitochondrial import receptor subunit TOM40 homolog 1 (Q9U4L6)|Evalue: 2e-90	contig00328:121:1038:1	EZ758957	1,15	1,97	1,11
	**protein transport**					
	similar to Mitochondrial import receptor subunit TOM20 homolog (A6H7B1)|Evalue: 3e-16	contig01412:1:447:1	EZ759155	2,16	/	/
	similar to Nucleoporin GLE1 (Q4KLN4)|Evalue: 3e-23	contig03033:1:1390:2	EZ759465	/	/	0,12
	similar to Protein transport protein Sec61 subunit alpha-like 1 (Q90ZM2)|Evalue: 1e-179	contig06044:1:1060:2	EZ759984	1,37	2,64	2,3
	|similar to Protein transport protein Sec61 subunit alpha-like 1 (Q90ZM2)|Evalue: 4e-64	contig27760:1:458:1	EZ763230	1,69	4,16	3,64
	similar to Ran-binding protein 17 (Q9H2T7)|Evalue: 2e-14	contig26298:1:343:3	EZ762993	0,84	0,52	/
	**L-glutamate transport**					
	similar to Mitochondrial glutamate carrier 1 (Q9D6M3)|Evalue: 3e-12	contig01826:1:238:2	EZ759231	/	1,6	1,05
	similar to Calcium-binding mitochondrial carrier protein Aralar1 (Q5RBC8)|Evalue: 4e-41	contig27059:1:452:3	EZ763123	/	0,45	0,75
	**mRNA/protein transport**					
	similar to Nuclear pore complex protein Nup88 (Q8CEC0)|Evalue: 1e-13	contig08274:1:2015:3	EZ760295	0,13	0,09	0,09
	**iron ion transport**					
	similar to Sideroflexin-3 (Q9JHY2)|Evalue: 3e-106	contig08407:69:1061:3	EZ760314	0,42	0,84	1,18
	similar to Sideroflexin-5 (Q925N0)|Evalue: 4e-99	contig25085:1:1011:1	EZ762713	/	0,5	0,19
	**ammonium transport**					
	similar to Ammonium transporter 1 member 2 (Q6K9G1)|Evalue: 2e-24	contig09658:1:461:2	EZ760471	0,84	/	/
	similar to Ammonium transporter 2 (Q9BLG3)|Evalue: 2e-05	contig24817:1:584:3	EZ762648	0,32	/	/
	**neurotransmitter transport**					
	similar to Excitatory amino acid transporter 1 (P24942)|Evalue: 5e-82	contig10433:1:1019:3	EZ760549	/	0,37	0,18
	**calcium ion transport**					
	similar to Plasma membrane calcium-transporting ATPase 4 (P23634)|Evalue: 1e-102	contig11608:1:909:2	EZ760667	0,62	0,84	0,63
	**sodium/potassium ion transport**					
	similar to Sodium/potassium-transporting ATPase subunit alpha (Q6RWA9)|Evalue: 4e-44	contig08413:1:307:1	EZ760316	/	1	2,1
	similar to Sodium/potassium-transporting ATPase subunit alpha (Q6RWA9)|Evalue: 2e-33	contig13779:85:487:1	EZ760836	0,46	3,18	2,57
	similar to Sodium/potassium-transporting ATPase subunit beta (P25169)|Evalue: 1e-52	contig26273:258:1214:3	EZ762985	0,38	1,99	2,01
	**zinc ion transport**					
	similar to Zinc transporter SLC39A7 (Q92504)|Evalue: 1e-08	contig22182:1:826:2	EZ761979	0,22	0,22	/
	**ATP synthesis coupled proton transport**					
	similar to V-type proton ATPase subunit S1 (P40682)|Evalue: 2e-05	contig21495:234:1124:3	EZ761884	0,55	0,4	0,3
	similar to ATP synthase subunit e, mitochondrial (Q00361)|Evalue: 4e-05	contig23516:1:322:2	EZ762290	2,48	0,64	0,64
	similar to ATP synthase subunit O, mitochondrial (Q2EN81)|Evalue: 8e-25	contig25813:1:416:2	EZ762887	2,09	5,79	3,61
	similar to ATP synthase subunit g, mitochondrial (Q5RFH0)|Evalue: 6e-10	contig22780:74:272:2	EZ762102	2,4	/	/
	**intracellular copper ion transport**					
	similar to Copper chaperone for superoxide dismutase (O14618)|Evalue: 5e-29	contig25087:1:383:2	EZ762714	0,56	0,56	0,5
	**sulfate transport**					
	similar to Sodium-independent sulfate anion transporter (Q86WA9)|Evalue: 3e-74	contig26145:1:1544:2	EZ762957	0,12	/	/
	similar to Mitochondrial dicarboxylate carrier (Q9QZD8)|Evalue: 2e-63	contig20802:1:732:1	EZ761786	/	0,59	/
	similar to Tricarboxylate transport protein, mitochondrial (P79110)|Evalue: 5e-107	contig26020:207:1118:3	EZ762936	/	0,6	0,43
	similar to Calcium-binding mitochondrial carrier protein Aralar1 (Q9VA73)|Evalue: 1e-70	contig26596:231:851:3	EZ763051	/	0,5	0,97
	**Total emPAI**			**19,22**	**29,99**	**24,79**
**Channel activity (MF, 5)**	**water transport**					
	similar to Aquaporin-9 (O43315)|Evalue: 8e-43	contig26144:1190:2191:2	EZ762956	/	0,18	/
	similar to Aquaporin-10 (Q96PS8)|Evalue: 3e-39	contig24282:1:693:1	EZ762494	/	0,26	/
	similar to Aquaporin-4 (Q5I4F9)|Evalue: 2e-36	contig17421:247:1275:1	EZ761061	15,82	5,52	6,56
	similar to Aquaporin-10 (Q96PS8)|Evalue: 4e-56	contig04424:252:1300:3	EZ759721	1,8	4,67	4,1
	similar to Aquaporin-3 (Q08DE6)|Evalue: 1e-39	contig01013:1:991:2	EZ759085	/	/	0,18
	**ion transport**					
	similar to Neuronal acetylcholine receptor subunit beta-2 (P09484)|Evalue: 3e-05	contig20272:1:245:1	EZ761697	1,28	3,08	7,14
	similar to Acetylcholine receptor subunit gamma (P02714)|Evalue: 9e-12	contig09320:131:802:2	EZ760421	0,26	1,72	2,1
	similar to Acetylcholine receptor subunit alpha-L1 (P23414)|Evalue: 4e-23	contig17705:184:1038:1	EZ761138	1,25	0,89	0,98
	similar to Neuronal acetylcholine receptor subunit eat-2 (Q9U298)|Evalue: 4e-08	contig02467:116:940:2	EZ759360	0,62	0,87	0,51
	similar to Acetylcholine receptor subunit alpha-like 2 (P17644)|Evalue: 7e-09	contig25495:79:789:1	EZ762811	/	0,42	0,54
	similar to Neuronal acetylcholine receptor subunit beta-3 (Q5IS75)|Evalue: 8e-10	contig19129:1:846:1	EZ761456	/	0,32	/
	similar to Acetylcholine receptor subunit gamma (P05376)|Evalue: 3e-14	contig01978:53:763:2	EZ759254	/	0,24	0,38
	similar to Neuronal acetylcholine receptor subunit alpha-4 (Q5IS77)|Evalue: 6e-11	contig25798:1:638:3	EZ762883	0,49	/	/
	similar to Neuronal acetylcholine receptor subunit alpha-6 (Q9R0W9)|Evalue: 7e-10	contig10452:143:865:2	EZ760553	0,26	/	/
	similar to Acetylcholine receptor subunit gamma (P02714)|Evalue: 9e-12	contig09320:131:802:2	EZ760421	/	/	/
	similar to Acetylcholine receptor subunit alpha (P02711)|Evalue: 7e-15	contig09059:1:888:1	EZ760388	0,2	/	/
	similar to Acetylcholine receptor subunit gamma (P05376)|Evalue: 5e-07	contig08448:42:872:3	EZ760321	0,33	/	/
	similar to Cullin-5 (Q29425)|Evalue: 3e-59	contig17293:1:839:3	EZ761050	0,34	0,32	/
	similar to Inositol 1,4,5-trisphosphate receptor type 1 (Q14643)|Evalue: 1e-84	contig04295:229:1159:1	EZ759699	0,18	0,09	0
	similar to Anoctamin-10 (Q4V8U5)|Evalue: 4e-126	contig19973:164:2185:2	EZ761629	/	0,12	0,16
	similar to Chloride intracellular channel exc-4 (Q8WQA4)|Evalue: 2e-50	contig26932:43:801:1	EZ763098	1,08	1,11	0,81
	similar to Voltage-dependent anion-selective channel protein 1 (Q60932)|Evalue: 4e-09	contig26155:1:131:3	EZ762959	7,25	20,41	12,9
	similar to Voltage-dependent anion-selective channel protein 2 (P81004)|Evalue: 2e-41	contig23401:118:845:1	EZ762252	10,78	22,08	18,67
	similar to Trimeric intracellular cation channel type B (Q7ZVP8)|Evalue: 4e-39	contig22692:232:1080:1	EZ762078	/	0,45	0,32
	**Total emPAI**			**41,94**	**62,75**	**55,35**
**Lipid transport (BP, 4)**	**Large lipid transporter protein superfamily**					
	similar to Apolipophorins (Q9U943)|Evalue: 7e-11	contig04531:1:1454:1	EZ759740	434,48	148,6	131,78
	similar to Apolipoprotein B-100 (P04114)|Evalue: 3e-18	contig24202:107:1017:2	EZ762460	106,16	47,92	45,38
	PREDICTED: similar to apolipoprotein B [Strongylocentrotus purpuratus] (XP_800206.2)|Evalue: 1e-07	contig18537:1:1312:2	EZ761306	141,61	65,75	55,45
	PREDICTED: apolipoprotein B [Danio rerio] (XP_694827.3)|Evalue: 2e-5	contig24531:1:590:3	EZ762575	162,83	38,74	55,8
	uncharacterized conserved protein [Glossina morsitans morsitans] (ADD18598.1)|Evalue: 1e-12	contig26593:129:609:3	EZ763050	1,08	1,76	0,98
	similar to Vitellogenin-1 (P87498)|Evalue: 3e-07	contig06595:1:952:2	EZ760059	206,56	107,89	97,94
	similar to Vitellogenin-2 (P05690)|Evalue: 2e-12	contig26360:1:1109:3	EZ763003	25,45	33,27	30,89
	similar to Vitellogenin-4 (P18947)|Evalue: 9e-11	contig06373:1:1480:1	EZ760037	107,82	60,6	56,55
	similar to Vitellogenin-4 (P18947)|Evalue: 5e-11	contig02294:1:661:1	EZ759328	65,94	95,06	73,72
	similar to Vitellogenin-6 (P18948)|Evalue: 4e-16	contig26295:1:531:2	EZ762991	76,29	37,64	40,05
	similar to Vitellogenin-6 (P18948)|Evalue: 5e-10	contig24586:1:771:2	EZ762594	34,26	55,07	60,25
	similar to Vitellogenin-6 (P18948)|Evalue: 5e-14	contig02293:1:648:1	EZ759327	219,67	134,37	113,52
	hypothetical protein BRAFLDRAFT_69973 [Branchiostoma floridae] (XP_002591410.1)|Evalue: 8e-04	contig02295:455:757:2	EZ759329	72,73	58,29	47,7
	similar to Microsomal triglyceride transfer protein large subunit (P55158)|Evalue: 4e-31	contig26982:1:1617:2	EZ763107	/	1,88	1,93
	**LDLR family (LDL binding proteins)**					
	similar to Very low-density lipoprotein receptor (P35953)|Evalue: 4e-24	contig23325:1:1472:3	EZ762231	1,15	0,12	0,12
	similar to Low-density lipoprotein receptor (P35951)|Evalue: 7e-05	contig19083:167:1503:2	EZ761443	0,36	1,67	1,52
	similar to Low-density lipoprotein receptor-related protein 5 (O75197)|Evalue: 1e-20	contig04458:347:1819:2	EZ759728	/	0,12	0,12
	similar to Very low-density lipoprotein receptor (P98155)|Evalue: 9e-21	contig25676:1:412:3	EZ762852	/	0,42	0,63
	similar to Low-density lipoprotein receptor-related protein 2 (A2ARV4)|Evalue: 2e-10	contig25250:813:1145:3	EZ762749	0,56	2,56	2,38
	**HDL binding proteins**					
	similar to Vigilin (Q8VDJ3)|Evalue: 2e-73	contig20907:1:1151:1	EZ761804	0,72	2,75	2,05
	similar to Vigilin (Q8VDJ3)|Evalue: 1e-76	contig23870:1:1221:3	EZ762387	0,49	1,85	1,38
	**perilipin family**					
	similar to Lipid storage droplets surface-binding protein 2 (Q9VXY7)|Evalue: 2e-08	contig18066:109:664:1	EZ761216	/	/	1,46
	similar to Perilipin-2 (Q9TUM6)|Evalue: 8e-15	contig23310:1:1017:1	EZ762224	1,52	0,29	0,29
	**Others**					
	similar to Aspartate aminotransferase, mitochondrial (P08907)|Evalue: 6e-64	contig18095:1:491:3	EZ761221	1,54	3,36	2,77
	similar to Phosphatidylinositol transfer protein alpha isoform (P48738)|Evalue: 6e-71	contig05263:1:701:3	EZ759863	0,77	2,56	0,98
	similar to Glycolipid transfer protein domain-containing protein 1 (Q6DBQ8)|Evalue: 7e-27	contig22720:1:938:3	EZ762086	/	0,43	0,31
	similar to Epididymal secretory protein E1 (P61918)|Evalue: 2e-16	contig18709:206:705:2	EZ761348	0,4	0,4	0,4
	**Total emPAI**			**1662,39**	**903,37**	**826,35**
**Lipid storage (BP, 5)**	**AGC Ser/Thr protein kinase family. RAC subfamily.**					
	similar to RAC serine/threonine-protein kinase (Q8INB9)|Evalue: 5e-52	contig11071:1:467:3	EZ760607	/	/	0,69
	similar to RAC serine/threonine-protein kinase (Q8INB9)|Evalue: 1e-123	contig11796:151:1318:1	EZ760691	0,63	0,75	0,63
	**perilipin family**					
	similar to Perilipin-2 (Q9TUM6)|Evalue: 8e-15	contig23310:1:1017:1	EZ762224	1,52	0,29	0,29
	**others**					
	similar to Ganglioside GM2 activator (Q60648)|Evalue: 5e-32	contig01739:211:885:1	EZ759213	0,72	0,26	/
	**Total emPAI**			**2,87**	**1,3**	**1,61**
**Nutrient reservoir activity (MF, 2)**	similar to Vitellogenin-6 (P18948)|Evalue: 5e-14	contig02293:1:648:1	EZ759327	219,67	134,37	113,52
	similar to Vitellogenin-4 (P18947)|Evalue: 5e-11	contig02294:1:661:1	EZ759328	65,94	95,06	73,72
	similar to Vitellogenin-1 (P87498)|Evalue: 3e-07	contig06595:1:952:2	EZ760059	206,56	107,89	97,94
	similar to Vitellogenin-6 (P18948)|Evalue: 5e-10	contig24586:1:771:2	EZ762594	34,26	55,07	60,25
	**Total emPAI**			**526,43**	**392,39**	**345,43**
**Lipid catabolic process (BP, 4)**	similar to Pancreatic lipase-related protein 2 (P54318)|Evalue: 2e-58	contig24738:99:1589:3	EZ762626	/	/	0,12
	similar to Pancreatic lipase-related protein 2 (Q64424)|Evalue: 5e-41	contig22166:1:1452:1	EZ761974	0,27	/	/
	similar to Group XV phospholipase A2 (Q8VEB4)|Evalue: 3e-59	contig22025:104:1296:2	EZ761946	0,24	/	0,9
	similar to Group 3 secretory phospholipase A2 (Q9NZ20)|Evalue: 5e-07	contig20744:89:1051:2	EZ761780	/	0,18	0,09
	similar to Gastric triacylglycerol lipase (Q9CPP7)|Evalue: 7e-53	contig20561:1:604:1	EZ761744	/	0,65	/
	similar to 85 kDa calcium-independent phospholipase A2 (O60733)|Evalue: 3e-121	contig11754:1:2273:3	EZ760687	/	0,16	/
	similar to Hepatic triacylglycerol lipase (P11150)|Evalue: 3e-34	contig11454:1:1130:3	EZ760647	1,22	/	/
	similar to Monoglyceride lipase (O35678)|Evalue: 3e-50	contig10509:753:1454:3	EZ760557	/	0,27	/
	similar to Phospholipase A-2-activating protein (Q6GM65)|Evalue: 1e-102	contig08576:1:1802:3	EZ760336	0,41	0,41	0,28
	similar to Phospholipase D3 (Q6PB03)|Evalue: 4e-101	contig07969:1:1665:1	EZ760255	/	0,1	/
	**Total emPAI**			**2,14**	**1,77**	**1,39**
**Defense response (BP, 3)**	similar to CD109 antigen (Q6YHK3)|Evalue: 3e-16	contig22765:1:560:3	EZ762099	1,54	17,92	15,09
	similar to Alpha-2-macroglobulin-like protein 1 (A8K2U0)|Evalue: 9e-05	contig14499:1:464:3	EZ760868	0,61	6,41	5,97
	similar to Pesticidal crystal protein cry8Ba (Q45705)|Evalue: 4e-05	contig26801:1:1803:1	EZ763078	4,03	2,18	1,79
	similar to Aminoacyl tRNA synthetase complex-interacting multifunctional protein 1 (P31230)|Evalue: 1e-40	contig25477:1:359:3	EZ763078	1,22	1,22	1,22
	similar to Pathogenesis-related protein 5 (P28493)|Evalue: 7e-35	contig05319:82:714:1	EZ759868	/	0,7	0,49
	similar to Histone H2B (P17271)|Evalue: 2e-44	contig17326:97:498:1	EZ761054	12,67	0,53	/
	similar to Transmembrane 9 superfamily member 4 (Q8BH24)|Evalue: 2e-179	contig09206:225:1888:3	EZ760404	/	0,2	0,16
	similar to CD109 antigen (Q6YHK3)|Evalue: 6e-36	contig18156:1490:2302:3	EZ761235	/	/	0,78
	similar to Thaumatin-like protein 2 (P83335)|Evalue: 3e-17	contig22647:1:758:3	EZ762068	/	/	0,25
	**Total emPAI**	Total emPAI		**20,07**	**29,16**	**25,75**
**Pathogenesis (BP, 3)**	similar to Ophanin (Q7ZT98)|Evalue: 5e-23	contig03806:1:678:1	EZ759615	1,68	/	0,27
	similar to Ophanin (Q7ZT98)|Evalue: 1e-24	contig08352:51:734:3	EZ760305	0,42	5,64	4,4
	similar to Pesticidal crystal protein cry8Ba (Q45705)|Evalue: 4e-05	contig26801:1:1803:1	EZ763078	4,03	2,18	1,79
	similar to Pathogenesis-related protein 5 (P28493)|Evalue: 4e-19	contig02384:1:343:2	EZ759345	/	/	1,04
	similar to Pathogenesis-related protein 5 (P28493)|Evalue: 1e-19	contig03028:130:858:1	EZ759463	/	0,26	1
	similar to Pathogenesis-related protein 5 (P28493)|Evalue: 9e-15	contig24159:1:208:2	EZ762449	/	4,48	3,39
	similar to Pathogenesis-related protein 5 (P28493)|Evalue: 6e-15	contig25505:1:208:2	EZ762814	/	3,39	4,48
	**Total emPAI**	Total emPAI		**6,13**	**15,95**	**16,37**
**Chitin metabolic process (BP, 6)**	**glycosyl hydrolase 18 family (Chitinase class II subfamily)**					
	similar to Chitinase D (P27050)|Evalue: 1e-08	contig28310:68:610:2	EZ763332	/	1,15	1,51
	similar to Endochitinase (P36362)|Evalue: 1e-41	contig26897:19:1285:1	EZ763092	/	0,75	0,58
	**glycosyl hydrolase 19 family. Chitinase class I subfamily.**					
	similar to Acidic endochitinase SP2 (P42820)|Evalue: 3e-16	contig22564:1:404:3	EZ762053	2,13	7,35	12,14
	**glycosyl hydrolase 19 family. Chitinase class IV subfamily.**					
	similar to Chitinase 5 (Q7Y1Z0)|Evalue: 2e-19	contig20760:1:720:3	EZ761781	/	1,6	0,84
	**glycosyl hydrolase 29 family.**					
	similar to Plasma alpha-L-fucosidase (Q6AYS4)|Evalue: 2e-52	contig28279:1:702:1	EZ763323	/	2,06	2,21
	similar to Plasma alpha-L-fucosidase (Q5RFI5)|Evalue: 4e-09	contig05782:1:148:1	EZ759937	/	2,38	1,95
	similar to Plasma alpha-L-fucosidase (Q6AYS4)|Evalue: 2e-125	contig05498:1:1566:3	EZ759895	/	0,22	0,15
	**Others**					
	PREDICTED: similar to ENSANGP00000013458 [Nasonia vitripennis] (XP_001599617.1)|Evalue: 7e-06	contig01202:555:721:3	EZ759118	7,02	12,14	14,71
	AGAP009479-PA [Anopheles gambiae str (XP_001230737.2)|Evalue: 1e-17	contig06721:1:451:2	EZ760080	3,64	4,8	4,48
	AGAP009479-PA [Anopheles gambiae str (XP_001230737.2)|Evalue: 3e-20	contig00352:1:376:1	EZ758961	1,69	/	0,48
	GK18229 [Drosophila willistoni] (XP_002066317.1)|Evalue: 4e-35	contig12046:1:481:2	EZ760724	1,25	/	/
	conserved hypothetical protein [Culex quinquefasciatus] (XP_001865643.1)|Evalue: 1e-18	contig04768:1:376:2	EZ759775	1,08	1,08	2,14
	hypothetical protein sce4008 [Sorangium cellulosum ‘So ce 56’] (YP_001614648.1)|Evalue: 7e-58	contig03173:1:473:1	EZ759493	1,02	/	0,7
	AGAP009479-PA [Anopheles gambiae str (XP_001230737.2)|Evalue: 2e-22	contig18379:1:612:3	EZ761274	1	6,58	4,39
	AGAP008123-PA [Anopheles gambiae str (XP_317336.3)|Evalue: 7e-74	contig12042:1:796:2	EZ760722	0,83	/	/
	PREDICTED: similar to CG14608 CG14608-PB, partial [Acyrthosiphon pisum] (XP_001942936.1)|Evalue: 3e-15	contig11457:1:1039:2	EZ760648	0,72	/	/
	chitinase A [Pteris ryukyuensis] (GI_110556116)|Evalue: 3e-05	contig07753:47:529:2	EZ760229	0,43	1,34	1,57
	hypothetical protein Bm1_29410 [Brugia malayi] (XP_001897324.1)|Evalue: 4e-15	contig07690:429:1916:3	EZ760219	0,18	/	0,12
	PREDICTED: similar to CG14301-PA isoform 1 [Apis mellifera] (XP_392551.2)|Evalue: 1e-17	contig18676:129:680:3	EZ761342	/	0,58	1,3
	hypothetical protein Phum_PHUM355660 [Pediculus humanus corporis] (XP_002427954.1)|Evalue: 9e-18	contig01761:290:940:2	EZ759217	/	0,56	0,52
	AGAP009479-PA [Anopheles gambiae str (XP_001230737.2)|Evalue: 9e-20	contig06213:292:1380:1	EZ760014	/	0,34	0,43
	conserved hypothetical protein [Culex quinquefasciatus] (XP_001865643.1)|Evalue: 1e-12	contig13408:1:955:1	EZ760815	/	/	0,18
	PREDICTED: similar to CG14608-PA [Apis mellifera] (XP_395554.2)|Evalue: 3e-20	contig27723:142:903:1	EZ763221	/	0,25	0,99
	GH19216 [Drosophila grimshawi] (XP_001990223.1)|Evalue: 1e-17	contig07253:7:561:1	EZ760163	/	1,3	1,24
	conserved hypothetical protein [Culex quinquefasciatus] (XP_001865643.1)|Evalue: 1e-12	contig13408:1:955:1	EZ760815	/	0,27	/
	chitinase A [Equisetum arvense] (GI_257074554)|Evalue: 1e-05	contig17011:111:854:3	EZ761013	/	0,56	0,73
	**Total emPAI**			**20,99**	**45,31**	**53,36**
**Embryonic development ending in birth or egg hatching (BP, 4)**						
	similar to AP-2 complex subunit sigma (Q17QC5)|Evalue: 3e-64	contig02168:1:786:1	EZ759301	0,22	/	/
	similar to ADP-ribosylation factor 1 (P61210)|Evalue: 5e-97	contig02660:89:739:2	EZ759392	1,44	/	0,89
	similar to ADP-ribosylation factor-like protein 8A (Q96BM9)|Evalue: 1e-48	contig06765:39:331:3	EZ760088	1,2	0,69	0,69
	similar to Vitellogenin-4 (P18947)|Evalue: 9e-11	contig06373:1:1480:1	EZ760037	107,82	60,6	56,55
	similar to Vitellogenin-4 (P18947)|Evalue: 5e-11	contig02294:1:661:1	EZ759328	65,94	95,06	73,72
	similar to Vitellogenin-2 (P05690)|Evalue: 2e-12	contig26360:1:1109:3	EZ763003	25,45	33,27	30,89
	similar to Vitellogenin-6 (P18948)|Evalue: 4e-16	contig26295:1:531:2	EZ762991	76,29	37,64	40,05
	similar to Probable pyruvate dehydrogenase E1 component subunit alpha, mitochondrial (P52899)|Evalue: 7e-140	contig06986:298:1467:1	EZ760122	0,34	1,92	1,91
	similar to Pyruvate dehydrogenase E1 component subunit beta, mitochondrial (O44451)|Evalue: 1e-135	contig25872:119:1225:2	EZ762900	0,48	2,12	2,37
	similar to Probable nuclear transport factor 2 (Q21735)|Evalue: 3e-27	contig25782:1:501:1	EZ762878	2,17	/	/
	similar to Cytochrome c oxidase subunit 5A, mitochondrial (P55954)|Evalue: 2e-27	contig18666:1:338:3	EZ761338	1,32	/	/
	similar to Histone H3.2 (Q5MYA4)|Evalue: 3e-52	contig07813:1:482:3	EZ760238	0,82	/	/
	similar to Histone H3.3 (P84247)|Evalue: 2e-73	contig06818:1:834:1	EZ760098	1,77	/	/
	similar to Histone H1-delta (P15870)|Evalue: 2e-09	contig02313:281:1393:2	EZ759334	0,34	/	/
	similar to Plasma membrane calcium-transporting ATPase 3 (Q16720)|Evalue: 1e-67	contig06386:752:1527:2	EZ760038	0,24	1,44	1,03
	similar to Serine/threonine-protein phosphatase PP1-beta (Q627N3)|Evalue: 2e-174	contig17157:152:1162:2	EZ761034	1,69	0,59	1,86
	similar to Ras-related protein Rab-6.2 (Q22782)|Evalue: 4e-31	contig05996:1:211:3	EZ759974	1,52	/	/
	similar to Probable V-type proton ATPase 116 kDa subunit a (P30628)|Evalue: 2e-28	contig08886:1:238:1	EZ760368	0,74	/	0,74
	similar to 40S ribosomal protein S14 (P48150)|Evalue: 2e-57	contig05614:107:562:2	EZ759912	7,18	1,82	2,84
	similar to 60S ribosomal protein L9 (O02376)|Evalue: 1e-11	contig20627:1:208:2	EZ761755	6,79	6,31	6,55
	similar to Tubulin alpha-2 chain (P34690)|Evalue: 2e-59	contig05639:1:407:3	EZ759917	/	0,82	/
	similar to Probable eukaryotic translation initiation factor 3 subunit G (A8WLV5)|Evalue: 4e-16	contig06785:1:330:1	EZ760090	1,09	1,54	0,56
	similar to Protein unc-112 (Q18685)|Evalue: 1e-38	contig11158:129:893:3	EZ760617	0,24	1,03	0,72
	similar to Thiol protease aleurain (P05167)|Evalue: 1e-10	contig25969:1:208:2	EZ762922	/	2,01	2,22
	similar to Dolichyl-diphosphooligosaccharide–protein glycosyltransferase subunit dad-1 (P52872)|Evalue: 1e-40	contig21689:47:381:2	EZ761915	0,54	0,81	0,81
	similar to Developmentally-regulated GTP-binding protein 1 (Q9Y295)|Evalue: 5e-34	contig05742:1:412:2	EZ759929	1,08	/	/
	similar to Developmentally-regulated GTP-binding protein 1 (Q9Y295)|Evalue: 7e-82	contig21323:1:561:1	EZ761860	1,06	0,78	1,4
	similar to Elongation factor 1-alpha (P41752)|Evalue: 0.0	contig22711:32:1325:2	EZ762083	32,72	38,85	44,47
	similar to Elongation factor 2 (Q96X45)|Evalue: 4e-124	contig11452:1:1044:1	EZ760646	/	0,27	0,18
	similar to Eukaryotic initiation factor 4A (P27639)|Evalue: 4e-136	contig22755:88:1314:1	EZ762095	0,46	0,46	0,74
	similar to Guanine nucleotide-binding protein G(o) subunit alpha (P51877)|Evalue: 0.0	contig25108:137:1204:2	EZ762722	0,36	1,03	0,47
	similar to Inorganic pyrophosphatase (O77460)|Evalue: 1e-31	contig18740:1:458:1	EZ761357	/	1,2	1,01
	similar to Integrin beta pat-3 (Q27874)|Evalue: 1e-63	contig03490:1845:2819:1	EZ759555	/	0,29	
	similar to Integrin-linked protein kinase (Q5R5V4)|Evalue: 9e-164	contig01873:1:1761:1	EZ759237	/	0,69	0,69
	similar to NADH dehydrogenase [ubiquinone] 1 alpha subcomplex subunit 13 (Q95KV7)|Evalue: 7e-17	contig08535:1:521:3	EZ760333	0,51	/	/
	similar to Myosin-3 (P12844)|Evalue: 4e-08	contig00571:1:108:2	EZ759003	/	31,51	66,84
	similar to N-terminal acetyltransferase complex ARD1 subunit homolog B (Q9BSU3)|Evalue: 8e-67	contig03086:118:795:1	EZ759477	0,8	0,68	0,38
	similar to 6-phosphogluconate dehydrogenase, decarboxylating (P41570)|Evalue: 0.0	contig17746:1:1516:2	EZ761148	4,6	4,23	3,76
	similar to Protein disulfide-isomerase 2 (Q17770)|Evalue: 6e-144	contig26565:106:1698:1	EZ763042	14,61	36,48	30,18
	similar to Lupus La protein homolog A (P28048)|Evalue: 1e-22	contig20066:1:1250:3	EZ761650	0,72	0,72	1,1
	similar to Staphylococcal nuclease domain-containing protein 1 (Q7ZT42)|Evalue: 1e-54	contig11006:1:669:1	EZ760602	0,86	4,71	2,19
	similar to Succinyl-CoA ligase [ADP-forming] subunit beta (A7HT39)|Evalue: 1e-09	contig24470:1:155:3	EZ762557	5,25	7,63	5,25
	similar to Surfeit locus protein 4 homolog (Q18864)|Evalue: 2e-80	contig23840:437:1324:2	EZ762378	/	0,2	0,41
	similar to Threonyl-tRNA synthetase, cytoplasmic (P52709)|Evalue: 5e-96	contig27426:1:751:1	EZ763176	0,25	0,87	0,37
	similar to Transcription factor BTF3 homolog (Q18885)|Evalue: 6e-25	contig23482:88:621:1	EZ762279	2,15	3,4	1,6
	similar to Translocon-associated protein subunit alpha (P43307)|Evalue: 5e-30	contig24346:41:685:2	EZ762511	1,12	1,9	1,3
	similar to Transmembrane protein 33 homolog (Q9XWV0)|Evalue: 3e-30	contig24567:2523:3216:2	EZ762587	0,4	0,74	/
	similar to Collagen alpha-2(IV) chain (P27393)|Evalue: 2e-88	contig05223:1:537:1	EZ759853	/	2,3	1,85
	similar to Aminoacyl tRNA synthetase complex-interacting multifunctional protein 1 (O54873)|Evalue: 1e-07	contig04349:1:280:1	EZ759712	0,7	0,7	0,7
	similar to Ubiquitin-conjugating enzyme E2-17 kDa (P25867)|Evalue: 2e-52	contig01677:1:482:1	EZ759203	0,6	/	/
	similar to 2,3-bisphosphoglycerate-independent phosphoglycerate mutase (Q0STD7)|Evalue: 4e-135	contig12053:1:1528:2	EZ760725	0,33	1,75	1,54
	**Total emPAI**			**374,21**	**389,06**	**390,83**

BP: biological process; MF: molecular function.

To analyze the major components in each state we selected protein hits which showed an emPAI of >30. We found 38 proteins as major components in EES, from which 20 are without annotation (Table 2). Among annotated proteins we found 10 protein members of the large lipid transporter protein (LLTP) superfamily [18] such as apolipophorins and vitellogenins. Heat shock proteins and ribosomal proteins are further proteins of the major component category. 60S ribosomal protein L7 and 40S ribosomal proteins S30 are involved in translation and in particular 60S ribosomal protein L7 is known to be involved in reproduction and embryonic development ending in birth or egg hatching [19]. Two heat shock proteins are present: Hsc71 and sHsp p40 (major egg antigen) which is highly expressed in EES. Furthermore we found only one protein belonging to structural constituent of cytoskeleton (actin-5C). Other cytoskeleton proteins seem to be not highly expressed at this state.

**Table 2:** Major protein components in early embryonic state (EES).

Protein description	NCBInr accession no.	Protein length (aa)	Slice no.	emPAI
contig18794:1:101:3|No Annotation*	EZ761369	33	EES1, 3	874.69
contig18438:8:349:2|No Annotation*	EZ761288	113	EES9, 1–24	535.33
contig04531:1:1454:1|similar to Apolipophorins (Q9U943)|Evalue: 7e-11	EZ759740	484	EES5, 1–25	434.48
contig08625:1:110:2|No Annotation	EZ760340	36	EES8, 2–22	275.13
contig10105:1:309:3|No Annotation	EZ760515	102	EES6, 1–23	258.84
contig18673:1:499:2|similar to Major egg antigen (P12812)|Evalue: 2e-05	EZ761340	166	EES9, 1, 3–23	227.19
contig02293:1:648:1|similar to Vitellogenin-6 (P18948)|Evalue: 5e-14	EZ759327	216	EES6, 2–23, 27	219.67
contig06595:1:952:2|similar to Vitellogenin-1 (P87498)|Evalue: 3e-07	EZ760059	317	EES6, 1–25	206.56
contig08235:860:1596:2|No Annotation*#	EZ760287	245	EES12, 3–24	197.64
contig24531:1:590:3|PREDICTED: apolipoprotein B [Danio rerio](XP_694827.3)|Evalue: 2e-05	EZ762575	196	EES5, 1–23	162.83
contig18537:1:1312:2|PREDICTED: similar to apolipoprotein B [Strongylocentrotus purpuratus] (XP_800206.2)|Evalue: 1e-07	EZ761306	437	EES5, 1–22, 24	141.61
contig26443:1:303:1|No Annotation	EZ763018	101	EES8, 1–20	126.67
contig06373:1:1480:1|similar to Vitellogenin-4 (P18947)|Evalue: 9e-11	EZ760037	493	EES6, 1–24	107.82
contig24202:107:1017:2|similar to Apolipoprotein B-100 (P04114)|Evalue: 3e-18	EZ762460	303	EES5, 1–24	106.16
contig13035:1:170:1|No Annotation	EZ760790	56	EES10, 2–21	90.14
contig26295:1:531:2|similar to Vitellogenin-6 (P18948)|Evalue: 4e-16	EZ762991	176	EES6, 1–20	76.29
contig02295:455:757:2|No Annotation	EZ759329	101	EES6, 3–23	72.73
contig19498:1:162:1|No Annotation	EZ761534	53	EES17, 16–18	69.57
contig23734:1:153:2|similar to Transketolase-like protein 2(Q9D4D4)|Evalue: 6e-16	EZ762348	50	EES8, 8–11, 13–16	66.59
contig02294:1:661:1|similar to Vitellogenin-4 (P18947)|Evalue: 5e-11	EZ759328	220	EES6, 4–22	65.94
contig21262:473:652:1|No Annotation	EZ761852	59	EES18, 1, 4, 16–21	60.53
contig08235:1:820:2|No Annotation	EZ760287	272	EES12, 3–21, 23	57.82
contig24360:1:563:3|No Annotation	EZ762516	187	EES6, 3–20	54.9
contig21510:1907:2014:1|similar to 40S ribosomal protein S30(P62861)|Evalue: 3e-05	EZ761888	35	EES20, 1, 21	43.57
contig02022:1:119:3|No Annotation	EZ759264	39	EES13, 3–11, 12–15	41.58
contig28231:1:506:3|No Annotation	EZ763311	168	EES8, 1–17, 20, 21, 23	40.43
contig13522:1:269:3|No Annotation	EZ760825	89	EES6, 1, 3, 5–17, 21	39.96
contig08851:1:613:2|similar to 60S ribosomal protein L7 (O01802)|Evalue: 4e-82	EZ760361	203	EES1, 1–4	39.62
contig20910:105:416:3|similar to Histone H4 (P62799)|Evalue: 9e-40	EZ761805	103	EES20, 1–5, 14–24	37.24
contig03062:1:281:2|No Annotation	EZ759470	93	EES6, 3–22	37.22
contig17982:1:724:2|similar to Actin-5C (P10987)|Evalue: 1e-138	EZ761195	240	EES12, 13	36.13
contig18941:155:934:2|No Annotation	EZ761416	259	EES14, 1, 11–21	35.41
contig02694:1:571:2|No Annotation*	EZ759400	189	EES10, 6–20	34.64
contig24586:1:771:2|similar to Vitellogenin-6 (P18948)|Evalue: 5e-10	EZ762594	256	EES6, 3–20	34.26
contig26339:1:396:1|No Annotation	EZ762998	131	EES15, 14–16	33.54
contig22711:32:1325:2|similar to Elongation factor 1-alpha (P41752)|Evalue: 0.0	EZ762083	431	EES10, 6–21	32.72
contig07785:1:816:1|No Annotation	EZ760233	272	EES6, 2–17	31.07
contig07915:1:1267:2|similar to Heat shock cognate 71 kDa protein(Q90473)|Evalue: 0.0	EZ760247	422	EES8, 7–20	30.43

38 proteins were found as major components in EES, 10 of which belong to the large lipid transporter superfamily. Other members are the heat shock protein family, structural constituent of ribosome and cytoskeleton. In addition, 20 proteins are without annotation. The contig description is indicated with asterisk, in case we found putative candidates by DomainSweep analysis. For one contig DomainSweep analysis delivered a significant candidate (indicated with #). Proteins with annotation are ordered by their biological function.

Proteins without annotation are indicated with an asterisk, in case we found putative candidates in DomainSweep results. For one contig (EZ760287/contig08235:1:820:2) DomainSweep analysis delivered a significant candidate (indicated with #), namely the whey acidic protein (WAP) 4-disulfide core. This protein has a peptidase inhibitor activity.

Contig18794:1:101:3 (EZ761369) contains only 33 amino acids and shows a high emPAI of 874.69 in EES. Generally, proteins with short sequences deliver a small number of observable peptides resulting in high emPAI values [17], [20]. On the other hand we have performed a relative comparative analysis of different states using the same database. The emPAI of this contig is considerably lower in AS (70.62) and TS (29.55), which means that the high emPAI is in fact due to the higher abundance in EES than AS or TS. Blast search of this contig against NCBInr delivered ribosomal protein L4 (*Danio rerio*), however with an insufficient e-value. DomainSweep analysis of this contig resulted in ribosomal protein L4/L1e as putative candidate. In-depth proteomics analysis is needed to verify these results.

We found 53 proteins as major components in adult tardigrades in AS and 49 in TS (Table 3). Comparing the annotated proteins in AS and TS we found the same three major functional groups, members of structural constituent of cytoskeleton and muscle, furthermore members of LLTP superfamily. Proteins without annotation include contigs (indicated with asterisk), for which we have found putative candidates by DomainSweep analysis.

**Table 3:** Major protein components in adult tardigrades in active and tun state. 53 proteins were found as major components in adult tardigrades in AS and 49 in TS.

Protein annotation	NCBInr accession no.	Protein length (aa)	Slice no. (AS)	emPAI (AS)	Slice no. (TS)	emPAI (TS)
contig18438:8:349:2|No Annotation*	EZ761288	113	AS6, 4–19	164.93	TS6, 4–19	431.91
contig08625:1:110:2|No Annotation	EZ760340	36	AS7, 4–20	175.51	TS7, 4–20	208.2
contig06212:1:430:3|No Annotation*	EZ760013	142	AS15	85.41	TS14, 10–19	150.89
contig04531:1:1454:1|similar to Apolipophorins (Q9U943)|Evalue: 7e-11	EZ759740	484	AS5, 2–20	148.6	TS5, 4–20	131.78
contig02293:1:648:1|similar to Vitellogenin-6 (P18948)|Evalue: 5e-14	EZ759327	216	AS7, 4–19	134.37	TS7, 4–27	113.52
contig17982:1:724:2|similar to Actin-5C (P10987)|Evalue: 1e-138	EZ761195	240	AS13, 11–15, 17–19	192.6	TS13, 12–14, 18, 19	101.14
contig06595:1:952:2|similar to Vitellogenin-1 (P87498)|Evalue: 3e-07	EZ760059	317	AS6, 3–26	107.89	TS6, 4–26	97.94
contig18673:1:499:2|similar to Major egg antigen (P12812)|Evalue: 2e-05	EZ761340	166	AS11, 4–19	108.34	TS10, 4–19	97.24
contig22876:95:450:2|similar to Actin, cytoplasmic 1(P68142)|Evalue: 3e-67	EZ762129	118	AS13, 4–8, 10–20	75.63	TS13, 1, 4–19	89.85
contig02294:1:661:1|similar to Vitellogenin-4 (P18947)|Evalue: 5e-11	EZ759328	220	AS7, 5–19	95.06	TS7, 4–20	73.72
contig00571:1:108:2|similar to Myosin-3 (P12844)|Evalue: 4e-08	EZ759003	35	AS12, 4, 6, 10, 14, 15, 17	31.51	TS7, 4, 6–9, 11, 12, 13	66.84
contig13522:1:269:3|No Annotation	EZ760825	89	AS7, 4–18	35.97	TS6, 4–19	66.72
contig22232:1:250:2|No Annotation	EZ761993	83	AS17, 16–19	33.65	TS17, 16–19	61.15
contig24360:1:563:3|No Annotation*	EZ762516	187	AS7, 4–17	60	TS7, 4–17	60.76
contig24586:1:771:2|similar to Vitellogenin-6(P18948)|Evalue: 5e-10	EZ762594	256	AS7, 4–19	55.07	TS7, 4–19	60.25
contig26443:1:303:1|No Annotation	EZ763018	101	AS7, 3–18, 20	54.39	TS7, 4–21	58.75
contig08235:860:1596:2|No Annotation#*	EZ760287	245	AS13, 4–19	68.46	TS13, 4–20	58.55
contig13035:1:170:1|No Annotation	EZ760790	56	AS9, 3–21	58.2	TS7, 4–21	58.5
contig23734:1:153:2|similar to Transketolase-like protein 2(Q9D4D4)|Evalue: 6e-16	EZ762348	50	not identified		TS9, 10, 12, 13, 16	57.59
contig06373:1:1480:1|similar to Vitellogenin-4 (P18947)|Evalue: 9e-11	EZ760037	493	AS6, 3–20	60.6	TS6, 2–21	56.55
contig24531:1:590:3|PREDICTED: apolipoprotein B [Danio rerio] (XP_694827.3)|Evalue: 2e-05	EZ762575	196	AS5, 3–19	38.74	TS5, 4–19	55.8
contig02694:1:571:2|No Annotation*	EZ759400	189	AS11, 6–20	40.57	TS11, 5–20	55.6
contig18537:1:1312:2|PREDICTED: similar to apolipoprotein B [Strongylocentrotus purpuratus] (XP_800206.2)|Evalue: 1e-07	EZ761306	437	AS5, 2–19	65.75	TS5, 4–19	55.45
contig14962:1:769:2|similar to Myosin heavy chain, muscle(P05661)|Evalue: 6e-58	EZ7692	255	AS5, 4–19	62.42	TS4, 4–17, 19	54.71
contig00982:1:961:2|similar to Actin-5C (P10987)|Evalue: 3e-137	EZ759080	319	AS13, 3–26	62.7	TS13, 4–25, 27	52.95
contig01345:1:144:1|similar to Myosin heavy chain, muscle(P05661)|Evalue: 2e-08	EZ759146	48	AS4, 4–11, 13–18	43.96	TS5, 4–17	51.66
contig02107:1:417:1|similar to Myosin heavy chain, muscle(P05661)|Evalue: 4e-35	EZ759286	139	AS8, 4–19	45.53	TS4, 4–19	49.63
contig10105:1:309:3|No Annotation	EZ760515	102	AS10, 4–20, 24	49.63	TS10, 4–19	49.34
contig24758:1:1673:3|similar to Paramyosin (Q86RN8)|Evalue: 2e-126	EZ762631	556	AS7, 4–19	62.28	TS7, 4–19	47.74
contig02295:455:757:2|No Annotation	EZ759329	101	AS7, 4–19	58.29	TS7, 4–19	47.7
contig02022:1:119:3|No Annotation	EZ759264	39	AS6, 4–17	43.8	TS7, 4–13, 15–17	46.92
contig24202:107:1017:2|similar to Apolipoprotein B-100(P04114)|Evalue: 3e-18	EZ762460	303	AS5, 4–20, 23, 24	47.92	TS5, 4–19	45.38
contig22711:32:1325:2|similar to Elongation factor 1-alpha(P41752)|Evalue: 0.0	EZ762083	431	AS11, 4–20	38.85	TS11, 4–20	44.47
contig25609:1:118:2|No Annotation	EZ762840	38	AS11, 10–16, 18	37.44	TS11, 10–16	41.76
contig05208:1:597:1|similar to Myosin heavy chain, muscle(P05661)|Evalue: 3e-50	EZ759850	199	AS8, 4–17	48.82	TS4, 4–14, 16, 17	41.35
contig26295:1:531:2|similar to Vitellogenin-6 (P18948)|Evalue: 4e-16	EZ762991	176	AS6, 4–19	37.64	TS7, 4–19	40.05
contig18052:1:207:1|No Annotation	EZ761213	69	AS8, 5–9, 16	35.33	TS9, 4, 8, 9, 16	39.78
contig07785:1:816:1|No Annotation	EZ760233	272	AS6, 4–19	48.7	TS7, 4–19	39.71
contig04301:1:1559:3|similar to ATP synthase subunit beta (Q39Q56)|Evalue: 0.0	EZ759701	518	AS11, 8–21	34.93	TS11, 10–21	39.26
contig26339:1:396:1|No Annotation*	EZ762998	131	AS17, 14–18	55.04	TS17, 15–18	36.42
contig20019:1:1092:1|similar to Arginine kinase (Q95V58)|Evalue: 1e-145	EZ761641	363	AS14, 12–23	37.1	TS14, 8, 12–20	35.73
contig00641:1:549:1|similar to Myosin heavy chain, striated muscle (P24733)|Evalue: 3e-50	EZ759017	183	AS6, 4–13, 15–18	34.33	TS4, 4–12, 15, 17	35.67
contig04802:115:695:1|No Annotation	EZ759786	193	AS11, 5, 7, 9–25	49.23	TS11, 10–23, 25, 26	34.66
contig13127:1:930:1|similar to Myosin-7 (Q91Z83)|Evalue: 1e-116	EZ760799	310	AS7, 2–18	46.57	TS4, 2, 4–15, 17–19	34.4
contig10543:1:889:2|similar to Filamin-A (Q8BTM8)|Evalue: 1e-72	EZ760561	295	AS8, 4–18	36.73	TS8, 4–18	32.72
contig20321:1:864:1|similar to 60S acidic ribosomal protein P0 (Q9U3U0)|Evalue: 8e-85	EZ761707	287	not identified		TS14, 14–20	31.26
contig26360:1:1109:3|similar to Vitellogenin-2 (P05690)|Evalue: 2e-12	EZ763003	368	AS9, 4–18	33.27	TS6, 4–19	30.89
contig26565:106:1698:1|similar to Protein disulfide-isomerase 2 (Q17770)|Evalue: 6e-144	EZ763042	530	AS10, 8–20	36.48	TS10, 9–20	30.18
contig00947:1:818:3|similar to Annexin A11 (P33477)|Evalue: 6e-48	EZ759075	271	not identified		TS14, 13–19	30.15
contig18794:1:101:3|No Annotation*	EZ761369	33	AS12, 12–19	70.62	not identified	
contig19607:1:514:2|No Annotation	EZ761554	171	AS6, 4–19	34.15	not identified	
contig23852:3162:3608:1|similar to Troponin I (P36188)|Evalue: 2e-23	EZ762384	148	AS16, 14–20	32.94	not identified	
contig07915:1:1267:2|similar to Heat shock cognate 71 kDa protein (Q90473)|Evalue: 0.0	EZ760247	422	AS9, 8–18	32.48	not identified	
contig01191:1:298:2|similar to 40S ribosomal protein S3 (Q90YS2)|Evalue: 2e-32	EZ759116	98	AS16, 4, 5, 12, 14–19	31.11	not identified	
contig26256:1:544:1|similar to Myosin heavy chain, muscle (P05661)|Evalue: 4e-37	EZ762982	181	AS6, 3–17, 19	30.15	not identified	
contig01971:138:399:3|AGAP000941-PA [Anopheles gambiae str (XP_560153.3)|Evalue: 8e-05	EZ759251	87	AS13, 11–16	30.05	not identified	

Comparing the annotated proteins in AS and TS we found the same two major functional groups, protein members of structural constituent of cytoskeleton/muscle and protein members of large lipid transporter family. The contig description is indicated with asterisk, in case we found putative candidates in DomainSweep analysis.

The same protein members of LLTP superfamily are present in AS as well as in TS. These include the following vitellogenin proteins: VTG-1, VTG-2, VTG-4 (2 different contigs), VTG-6 (3 different contigs). The early embryonic state contains all these vitellogenins except for vitellogenin-2. Interestingly, vitellogenin-2 is described to be involved in biological process of determination of adult lifespan, which means the control of viability and duration in the adult phase of the life-cycle [21], [22]. Actin 5-C (2 contigs), cytoplasmic actin and filamin-A belong to the structural constituent of cytoskeleton. Myosin heavy chain, paramyosin, myosin-7, myosin-3, and troponin I are muscle proteins and except troponin I have all motor activity function. We found one isoform of myosin heavy chain, troponin I, two protein members of heat shock protein family (Hsc 71 and AGAP000941-PA) and 40S ribosomal protein S3 as major components in AS, and annexin A11, transketolase-like protein 2 and 60S acidic ribosomal protein P0 as member of major component group in TS. Contig01971:138:399:3 is annotated as AGAP000941-PA from *Anopheles gambiae*, which shows high homology to small heat shock proteins. Among proteins without annotation, there are 18 proteins present in both AS and TS.

### Proteins Found in One State Only

**Figure 2:**
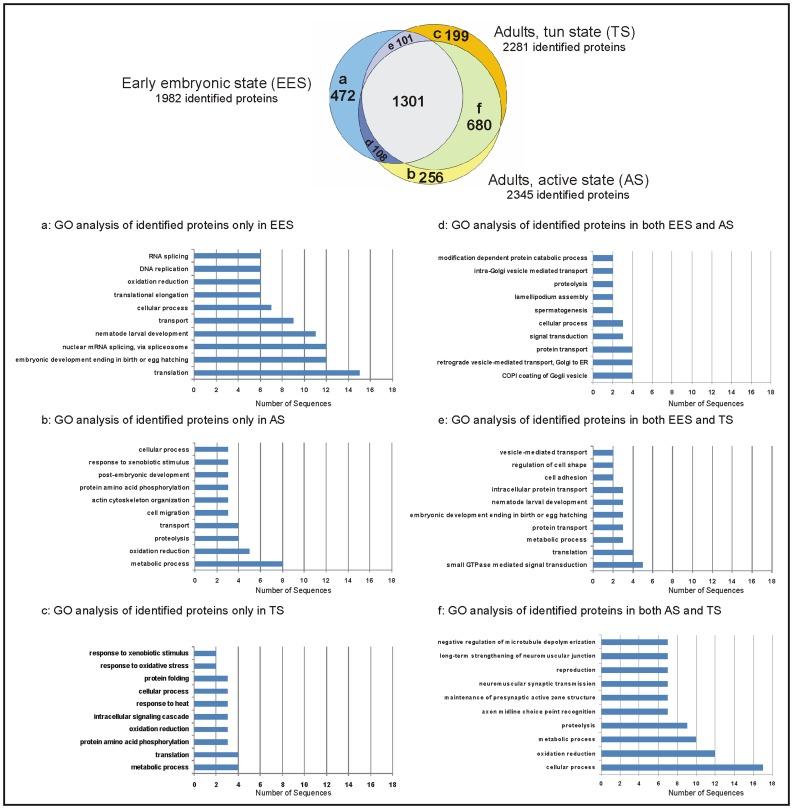
Comparative proteome analysis of proteins identified in different states. The Venn diagram illustrates the number of protein identifications in EES, AS and TS. A total of 1301 proteins were found in all three states, 472 proteins are found only in EES (a) and 680 proteins are found only in adult tardigrades in TS and AS (f). Proteins which are non-overlapping (a, b, c) or partially overlapping (d, e, f) between the different states are analyzed using Blast2GO program to determine the involved biological processes. The ten major biological processes for non-overlapping proteins are listed in 2a-2c and for partially overlapping proteins in 2d–2f.

The proteome analysis yielded 1982 proteins in EES, 2345 proteins in adult tardigrades in AS, and 2281 proteins in TS. A total of 1301 proteins are found in all three states as shown in the Venn diagram in Figure 2. 472 proteins are only identified in EES, 199 only in TS and 256 only in AS. To compare and demonstrate the main GO categories of biological process of proteins in single and overlapping regions (Venn diagram, a-f) Blast2GO program was applied. The highest ranked biological processes for each region are shown in Figure 2a-f.

A total of 472 proteins were identified only in EES, 122 of which are without annotation. Among the proteins identified only in EES, ribosomal proteins represent the majority as shown in Figure 2a. Although various ribosomal proteins are found in all states, there are 32 ribosomal proteins that are only observed in EES. The second main protein category present in EES contains proteins involved in embryonic development, which is expected (Figure 2a).

Specific proteins like protein members of the piwi family are identified only in EES. Piwi like proteins are developmental proteins that play a central role during gametogenesis [23]. Proteins involved in iron homeostasis like soma ferritin are also found only in EES. In total four contigs annotated as proteins belonging to ferritin family are identified (Table S4), two of which are present in all three states, one in EES and TS and another one only in EES. Two members of heat shock protein family are identified only in EES: the small heat shock protein C4 involved in stress response and 10 kDa heat shock protein belonging to the GroES chaperonin family and involved in protein folding. In addition two different contigs annotated as small heat shock protein major egg antigen (p40) are identified. One is only found in EES and the other one in all states.

A total of 256 proteins are found only in AS, from which 71 proteins are without annotation. The two proteins (EZ760543, EZ762990) with the highest emPAI value are without annotation and DomainSweep analysis delivered no specific protein domains. Dixin, a developmental protein involved in Wnt signalling pathway is the protein with the third highest emPAI value. Wnts control development in organisms ranging from nematodes to mammals. The Blast2GO analysis of annotated proteins (Figure 2b) delivered metabolic process, oxidation reduction and proteolysis as abundant categories, which are important processes for a living organism.

We identified 199 proteins in TS, from which 58 are without annotation. Two proteins without annotation (EZ758977, EZ762549) followed by myosin heavy chain (EZ763186) are the proteins with the highest emPAI value found only in TS. The result of Blast2GO analysis of annotated proteins is shown in Figure 2c. The first ten biological process categories include three categories involved in response to stimulus, such as heat, oxidative stress and xenobiotic stimulus (Figure 2c). Only the last one is also present in AS (Figure 2b). Heat shock protein 81-2 (Hsp90 family), hypoxia up-regulated protein 1 (Hsp70 family), and two members of DnaJ protein family are present as chaperones involved in stress response in tun state. Although activation of stress response was expected in TS, it seems there are other processes which are probably associated with anhydrobiosis. Proteins involved in intracellular signaling cascade and phosphorylation are present. Protein amino acid phosphorylation as a biological process category is present in Blast2GO results of proteins identified only in AS and TS (Figure 2b). However, the involved proteins in phosphorylation in both states seem to be different. Dual specifity mitogen-activated protein kinase (EZ759901/contig05524:314:1363:2), RAC serine/threonine-protein kinase (EZ760607) and cell division cycle 2-like protein kinase 6 (EZ761193), which are involved in phosphorylation have been identified only in TS.

Reanalysis of our data by including phosphorylation of serine, threonine and tyrosine as modification delivered 49 proteins (Table S5). We have identified 13 different phosphoproteins only in EES, 11 phosphoproteins only in AS and another 11 phosphoproteins only on TS. Further seven phosphoproteins are identified in both AS and TS, two phosphoproteins in both EES and AS and another two phosphoproteins in both EES and TS. We found 3 phosphoproteins in all three states. The comparison of the phosphoproteins which are found only in AS or TS shows that almost half of the phosphoproteins in TS are without annotation. Also of major interest are proteins involved in intracellular signaling cascade: calcium-regulated heat stable protein 1 (EZ759268), RAC serine/threonine-protein kinase (EZ760607) and Drebrin-like protein (EZ760971/contig16604:102:1247:3). However, the role of these proteins in relation to desiccation tolerance has to be investigated.

Among diverse proteins only identified in TS we found lipid storage droplets surface-binding protein which is involved in lipid transport and is reported to be required for normal deposition of neutral lipids in the oocytes [24], [25]. Lipids represent probably the only nutrient sources during all steps from dehydration (transitional state I) to rehydration (transitional state II) and thus are essential for surviving.

### Proteins Overlapping in Two States

Whereas 680 proteins were identified only in adult tardigrades (active and tun), the number of proteins which are overlapping between EES and adults is significantly lower (108 between EES and AS and 101 between EES and TS), which is expected (Venn diagram in Figure 2). Whereas cellular component organisation and transport are main processes in both EES and AS (Figure 2d), translation, development and biological regulation are abundant categories found in both EES and TS (Figure 2e). Proteins found only in AS and TS are mainly involved in cellular process, oxidation reduction, proteolysis and biological regulation (Figure 2f).

Proteins involved in metabolic processes are present in TS but reduced to half compared to AS, which is in accordance to the expectation since during anhydrobiosis a metabolic dormancy is described [26], [27].

## Discussion

### Comprehensive Analysis of the *M. tardigradum* Proteome

**Figure 3:**
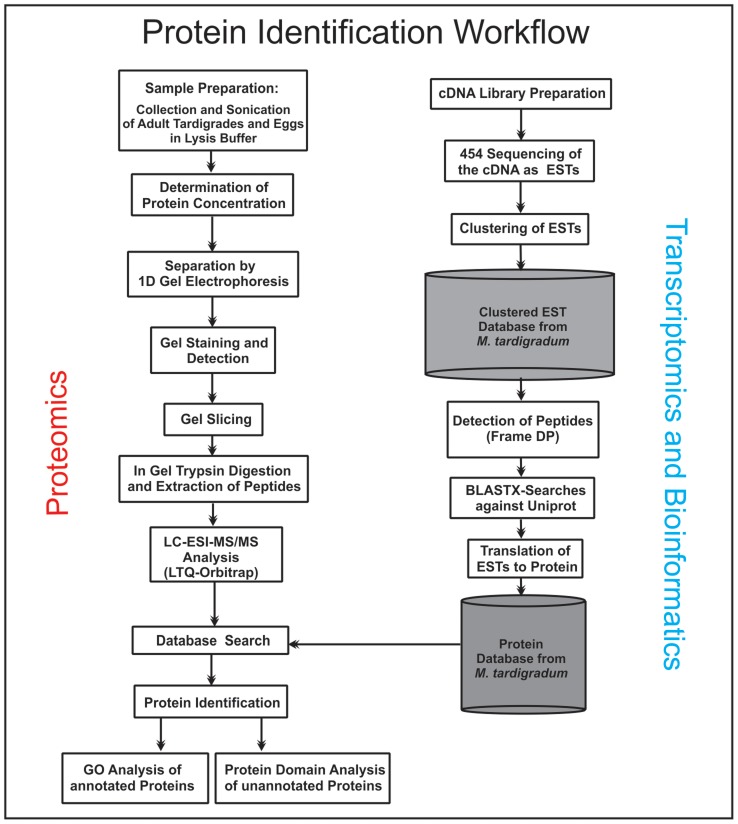
The experimental workflow to analyze the proteome of *Milnesium tardigradum*. Tardigrades in different states were homogenized directly in lysis buffer. Total protein extracts of tardigrades in early embryonic state and adult tardigrades in active and tun state were separated by 1D gel electrophoresis. After staining gel lanes were sliced and proteins in-gel digested with trypsin. MS/MS data obtained by nanoLC-ESI-MS/MS analysis were searched against the tardigrade specific database. The database was developed by translating EST sequences of *M. tardigradum*, which were obtained by 454 sequencing. Identified proteins with annotation were classified in different functional groups using the Blast2GO program. Identified proteins without annotation were analyzed with the DomainSweep program to search for specific protein domains.

In our previous publication a proteome map of tardigrades was developed utilizing 2D gel electrophoresis and LC-ESI-MS/MS analysis [15]. 2D gel electrophoresis offers high resolution and allows analysis of single spots, which contain at most only a few proteins. In particular, the absence of a comprehensive database at the time of our previous study made the reduction of complexity achieved by 2D gel electrophoresis necessary to increase the number of detected peptides which belong to the same protein. Since our parallel tardigrade EST sequencing project provided us recently with a high number of new EST sequences generated by 454 sequencing, we could consider the 1D gel electrophoresis as a complementary platform to 2D gel electrophoresis to analyze the proteome of tardigrades (Figure 3). The present study includes a comprehensive proteome resource of *M. tardigradum* and demonstrates the first comparative analysis of expressed proteins in three different states.

In addition we have reanalyzed the MS/MS data of protein spots from our 2D gel study [15] against the 454 database (Table S6). Interestingly, our 2D proteomics data of active tardigrades support the quantification analysis based on emPAI. Proteins with a high emPAI could be identified repeatedly in different protein spots on the 2D gel, which indicates the high amount of these proteins in the whole protein extract. For instance major egg antigen (EZ761340) shows a high emPAI of 108.34 and could be identified in 22 protein spots. Other proteins such as vitellogenin, apolipoproteins and actin show the same relation between emPAI and number of spots on the 2D gel.

Although the present 454 protein database is the most comprehensive one available at the moment, it is still an incomplete database. Calculation of emPAI using an incomplete database delivers high values for contigs with very short sequences, which can lead to misinterpretation [17], [20]. In these cases the high emPAI is caused by the calculation using a short sequence present in the database and therefore is not related to the amount of the protein. Nevertheless, a comparative analysis of the same protein in different states is possible, since we perform a relative quantification using the same database for all three states. In total we identified more than 3000 proteins, 2460 of which could be functionally annotated by homology search against the SwissProt and NCBInr databases. The results cover two main aspects:

- Identifcation of diverse protein families for the first time in *M. tardigradum.* Of major interest are proteins that have been reported to be related to anhydrobiosis such as heat shock proteins, Late Embryogenesis Abundant protein, aquaporins, and antioxidant proteins.
- Comparative analysis of major components in different states. Protein families identified only in early embryonic state deliver new aspects in terms of developmental biology. Comparative analysis of proteins in active *versus* tun state could bring us closer to understanding the molecular mechanisms during anhydrobiosis.

These two aspects are discussed in the following section by selected protein families.

### Comparative Analysis of Proteins Associated with Anhydrobiosis and Survival

Among the numerous proteins identified in this study some proteins have already been reported to play an important role during anhydrobiosis, most importantly Late Embryogenesis Abundant (LEA) proteins. Although the precise role of LEA proteins has not yet been fully elucidated, different studies have reported on the association of these proteins with tolerance to water stress by desiccation [28], [29]. The presence of LEA proteins in tardigrades has been shown by analyzing 2D gels prepared from whole protein lysates of *M. tardigradum* and homology search against NCBInr database [15]. In the present study LEA could be identified also in our tardigrade specific database. Contig EZ759004 shows high similarity to the LEA protein from *Alteromonas macleodii*. The predicted sequence from *M. tardigradum* was confirmed by MS/MS analysis of peptides covering 61.9% of the entire sequence (length: 147aa). This protein is up-regulated in adults and shows a 1.2 times higher emPAI in tun state compared to active state. The search for specific protein patterns using DomainSweep (Table S4) resulted in two significant hits (EZ759288, EZ761565) and 5 putative candidates (EZ759004, EZ759235, EZ761969, EZ762343, EZ762913) for LEA proteins. Among these candidates only contigs EZ759004, EZ759288 and EZ759235 are identified with more than one peptide.

Chaperones in particular heat shock proteins (Hsps) play key roles in cell protection and response to diverse stimuli like stress, heat and hypoxia by preventing protein aggregation (Table 1). The relation of Hsps in particular low molecular weight Hsps in desiccation tolerance and dormancy is reported in different studies [12], [30]. A comprehensive proteomic study of Hsps in tardigrades in active *versus* tun state has been reported earlier [16]. Different Hsp families are present in our results: Hsp90, Hsp70, Hsp60, Hsp40 and Hsp20, GroES, and GrpE families. We identified three sHsps that are described for the first time in *M. tardigradum*: the small heat shock protein C4 and 10kDa heat shock protein (GroES chaperonin family) identified only in the EES and a sHsp (AGAP000941-PA, sHsp 20.6 isoform 3 (EZ759251)) in all three states. In addition other chaperonin families such as TCP-1 and calreticulin were identified in all three states (Table S4). Our semi-quantitative analysis indicates an up-regulation of a small heat shock protein (major egg antigen, p40) and furthermore a ferritin homologue in EES of *M. tardigradum*. Major egg antigen is found in *Schistosoma mansoni* and is described to be involved in response to heat. In all analyzed states major egg antigen (EZ761340) is the heat shock protein with the highest emPAI, particularly in EES. Artemin, the ferritin homologue identified in *Artemia* is reported to protect cells from stress and acts similar to molecular chaperones such as small heat shock proteins. In studies on *Artemia* it has been shown that the small heat shock protein and artemin are associated with anhydrobiosis [31]. Since we found p40 and soma ferritin both up-regulated in EES and not in anhydrobiotic state, we assume that these proteins are involved in development and hence are specific markers for the EES. However, the role of p40 and ferritin in anhydrobiotic tardigrades has to be investigated.

**Figure 4:**
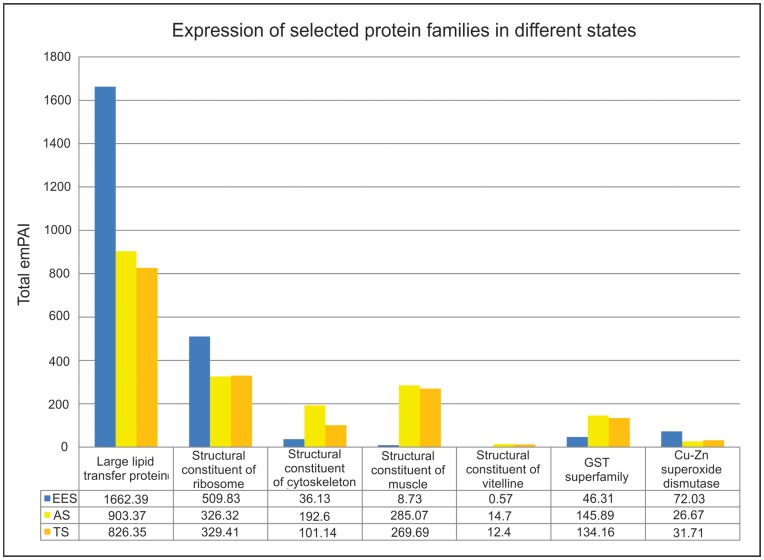
Comparative expression analysis of selected proteins. Total emPAI is the sum of emPAI of each protein member. Large lipid transfer protein (LLTP) superfamily belong to major components in all three states. This protein family is highly expressed in EES compared to adults. Semi-quantitative analysis of proteins contributing to the structural integrity of ribosome indicates a significant up-regulation of ribosomal proteins in EES. In contrast proteins involved in cytoskeletal-, muscle- and vitellin membrane structure are not highly abundant at this state compared to adults in AS and TS. Proteins with antioxidant activity such as GSTs are approximately 3 fold higher in adults compared to EES. In contrast Cu-Zn SODs are upregulated in EES.

An important aspect of desiccation tolerance is protection against free radicals [32], [33]. Superoxide dismutases (SODs) are one of the most important antioxidant enzymes in defense against ROS and particularly superoxide anion radicals [34], [35]. Generally SOD is present in two forms inside the eukaryotic cell, SOD (Cu-Zn) in the cytoplasm and outer mitochondrial space, and SOD (Mn) in the inner mitochondrial space [36]. Both superoxide dismutases SOD (Cu-Zn) (6 contigs) and SOD (Mn) (2 contigs) have been identified in tardigrades (Table S4). The superfamily of glutathione transferases (GSTs) builds a further cellular detoxification system [37]. In addition GSTs have cellular physiology roles such as regulators of cellular pathways of stress response and housekeeping roles in the binding and transport of specific ligands [38]. We have found 27 different contigs that belong to the GST superfamily. The expression of 1-cysteine (1-Cys) peroxiredoxin family of antioxidants is reported in *Arabidopsis thaliana* and is shown to be related to dormancy [39]. Different isoforms of peroxiredoxins (8 contigs) are included in our results. Peroxiredoxins and diverse other proteins like catalase, peroxidasin, thioredoxin reductase and glutamate cysteine ligase are described to be involved in response to oxidative stress (Table 1). The comparison of the total emPAI (sum of emPAI of each protein member) of protein families with antioxidant activity shows that GSTs are approximately 3 fold higher in adults compared to EES (Figure 4), which is probably due to the exposition to higher amounts of endobiotics and xenobiotics. Eggs are laid inside the old cuticle and remain there during the embryonic development. Therefore embryos are not directly attacked by xenobiotics. In contrast Cu-Zn SODs are up-regulated in EES compared to adults (Figure 4). The studies on development of mouse embryos *in vitro* have shown that thioredoxin and SODs promote the *in vitro* development of mouse embryos fertilized *in vitro* [40]. This suggests that protection of embryos from oxidative stress is a prerequisite for their development *in vitro*. We assume that the up-regulation of Cu-Zn SODs in EES is related to their important roles in development. Comparing active to tun state we observed up-regulation of GSTs and peroxiredoxins in active state and in contrast up-regulation of SODs in tun state.

We identified 350 transmembrane proteins, 53 of which are involved in transmembrane transport. One group of channel proteins that plays an important role in “desiccation-tolerance strategy” is the aquaporin (AQP) protein family [11]. AQPs are passive transport channels for water and permit water to move in the direction of an osmotic gradient. Kikawada et al. could show that AQP is involved in the removal of water in the desiccation process en route to anhydrobiosis [11]. Different AQPs are identified in *M. tardigradum*: AQP 3, AQP 4, AQP 9, AQP 10 (2 contigs). AQP 4 is the most abundant one in all three states and in particular up-regulated in EES. Compared to the active state AQP 4 is 1.2 times more expressed in the tun state. However, the question whether identified AQPs are involved in anhydrobiosis in *M. tardigradum* needs to be answered by performing functional analysis.

Although a high number of 1981 proteins overlaps between AS and TS, there are also numerous proteins (256 in AS and 199 in TS) that are identified only in one state. The Blast2GO analysis of proteins identified only in TS led to the assumption that not only the response to stimuli plays an important role in the anhydrobiotic state, but also further processes and mechanisms are associated such as response to heat, oxidative stress, intracellular signalling cascades, and phosphorylation. As shown in Table 1 we found not only Hsps involved in response to stimuli, but also two other main groups namely kinases in particular those involved in mitogen-activated (MAPK) signaling pathway and translation initiation factor, which is associated with protein biosynthesis. There are reports of observed changes in protein phosphorylation in plants which were exposed to water deficit, suggesting reversible phosphorylation as a regulator [41]. In particular mitogen-activated protein kinases (MAPKs) and other kinases belonging to the MAPK cascade have been identified in plants in response to dehydration, suggesting that the MAPK cascade is involved in stress signaling [42], [43].

The analysis for phosphorylation delivered 49 phosphoproteins (Table S5). As expected the number of identified phosphopeptides was very low, since we did not perform any enrichment steps for phosphopeptides prior to the mass spectrometry analysis. Enrichment steps need high amounts of peptides and have to be optimized. In our study the starting material was limited and did not allow any further procedures. However, we found specific phosphoproteins for each state. Almost half of the phosphoproteins in TS (5 out of 11) are without annotation. The functional analysis of these tardigrade specific proteins has to be investigated.

### Comparative Analysis of Proteins Identified in EES Versus AS and TS

Members of large lipid transfer protein (LLTP) superfamily belong to major components in all three states. In addition this superfamily is highly expressed in EES compared to adults as shown by calculating total emPAI (Figure 4). Lipid transport in animals is mediated by members of the LLTP superfamily, which are grouped into three major families: the apoB-like LLTPs, the vitellogenin-like LLTPs, and the microsomal triglyceride transfer protein (MTP)-like LLTPs, or MTPs [18]. In addition to lipid transport they have also been reported to play an important role in animal development [44], reproduction [22], and immunity [45] as well as aging and lifespan regulation [46]. The high regulation of protein members of LLTP superfamily in adults can be explained by the fact that we used middle age, egg producing adults in our experiments. ApoB-like LLTPs are represented in our study by the following protein members: three contigs which show high homology to apolipoprotein B, apolipoprotein O and apolipophorin (Table 1). Glycolipophosphoprotein vitellogenin (VTG) is the major precursor of the egg-yolk protein, vitellin (Vn), which provides sources of nutrients during embryonic development in oviparous organisms [22], [47]. It has been reported that lower vertebrates possess multiple Vtg genes and proteins [47] as has been shown for *Danio rerio* [48], *Xenopus laevis* [49], and salmonid fishes [50] as well as for the nematode *Caenorhabditis elegans* [51]. Similarly multiple Vtg proteins are found in *M. tardigradum*: VTG-1, VTG-2, VTG-4 (2 contigs) and VTG-6 (3 contigs). Whereas apoB-like LLTPs and vitellogenin-like LLTPs are present abundantly in EES, MTP-like LLTPs are underrepresented and are found only in adult tardigrades.

Other proteins could be identified that are associated with lipid transport and metabolism such as low-density lipoprotein receptors (LDLR family), vigilins (perilipin family) and high density lipoprotein-binding proteins. Also of interest are proteins associated with lipid catabolic process such as lipases. Lipoprotein lipases are assumed to be involved in fatty acid uptake, transport and metabolism. They are also known to serve as yolk proteins in dipterans eggs [52]. All protein categories related to lipid transport, storage and metabolism (Table 1) are significantly up-regulated in EES, since they most likely present a key source for energy during embryonic development. Similarly it has been shown that lipid metabolic pathways were up-regulated in the *C. elegans* dauer larval stage [53]. Furthermore association of these proteins with hibernation and dormancy can be expected since it is shown that lipids also serve as the main energy source in hibernating mammals [54]. Lipid metabolism associated proteins are almost similarly expressed in both AS and TS in tardigrades.

Other major components in EES are ribosomal proteins. This is also reflected in the semi-quantitative analysis of ribosomal proteins by comparing the total emPAI of all three states (Figure 4). Furthermore we found 32 ribosomal proteins that are only identified in EES. This result can be explained by the high need of protein synthesis with diverse functions including development en route to a mature organism. Proteins contributing to the structural integrity of cytoskeletal, muscle and vitelline membrane structure are weakly expressed in EES (Figure 4). Since vitelline membrane is a portion of egg shell, we expected the expression of those proteins only in mature animals, which is also reflected in our semi-quantitative analysis. Similarly proteins associated with pathogenesis such as pathogenesis-related protein 5 (PR-5, thaumatin family) are mainly expressed in adults. Eggs are laid inside the old cuticle and remain there during the embryonic development. Therefore developing embryos are not directly attacked by pathogens. The need of defence mechanisms against pathogens and fungi leads to higher expression of these proteins in adults (Table 1). Chitinases are widely distributed in a broad range of species and are described to be involved in digestion, arthropod molting, defence/immunity and pathogenicity by degrading of chitin and chitodextrins of chitin containing fungal pathogens (for review see [55]. Ophanin belonging to cysteine-rich secretory protein (CRISP) family is characterized as a snake venom protein that acts as a neurotoxin by targeting and inhibiting the voltage-gated calcium channels on smooth muscle [56]. Two contigs are annotated as ophanin; one is found only in EES and TS and the other one in all three states and is up-regulated in AS. Since adult tardigrades are carnivorous we assume that ophanin has not only defence function but is also used to trap the prey animals.

### Conclusion

The current study presents the first comparative proteome analysis of tardigrades in different states, which is an important resource for future research in this area. Since the amount of biological material was highly limited we were not able to perform biological replicates. However, the main focus of our study was to obtain information of highly abundant protein families present in the different life states of tardigrades rather than an accurate quantification of differentially expressed proteins. The semi-quantitative analysis of proteins served predominantly for estimation of relative protein concentration to grouping the proteins into minor and major components. This method mainly delivered results in comparing EES with adult animals in AS and TS. The up-regulation of specific protein families such as large lipid transfer (LLTP) superfamily and ribosomal proteins in EES could clearly be demonstrated. However, since the majority of 1981 unique proteins overlapped between AS and TS there is a need to extend the applied label-free quantification method to other more accurate techniques such as labeling-based approaches to detect even subtle differences in protein expression between AS and TS. For selecting the suitable quantification technology there are some limitations. Technologies such as SILAC [57] and ^14^N*/* ^15^N [58] metabolic labeling rely on metabolic incorporation of heavy isotopes and are suitable for cell culture and only in rare cases for whole organism [59] because the whole food chain of the organism has to be considered for labeling. Furthermore the number of tardigrades cultivated in the laboratory is limited and only the homogenization of a high number of individuals results in enough protein amount to perform experiments with biological replicates. This represents the major limitation in investigating tardigrades and makes quantification a challenging task.

## Materials and Methods

### Tardigrade Culture and Sampling

Tardigrades of the species *Milnesium tardigradum* Doyère (1840) were obtained from Dr. Ralph O. Schill (Department of Zoology, University of Stuttgart, Stuttgart, Germany) as described in our previous study and were maintained in a laboratory culture [12]. Briefly, the culture was grown on agarose plates (3%) (peqGOLD Universal Agarose, peqLAB, Erlangen Germany) covered with Volvic™ water (Danone Waters, Wiesbaden, Germany) at 20°C. The juveniles were fed on green algae *Chlorogonium elongatum*, the adults with bdelloid rotifers *Philodina citrina*. The specimens for the experiments were all of middle-age (egg producing), thus effects of age can be excluded. Tardigrades were starved for 3 days before harvesting and washed several times with Volvic™ water to avoid contamination with food-organisms. Subsequently the animals were transferred to microliter tubes (200 individuals per tube) and surrounding water was reduced to approx. 1–2 µl. Active (I) and anhydrobiotic states (III) according to Schill et al. [12] and eggs in the early embryonic state (blastula state), according to Suzuki [2] were investigated in this study. For the induction of the anhydrobiotic state (III), animals were desiccated in open microliter tubes (Biosphere SafeSeal Micro Tubes, Sarstedt, Nümbrecht, Germany) exposed to 85% relative humidity (RH) in a chamber containing a saturated solution of KCl (Roth, Karlsruhe, Germany) at 21°C for 24 h, subsequently transferred to a chamber containing a saturated MgCl_2_ solution (Roth, Karlsruhe, Germany), where they were exposed to 33% RH for at least 48 h.

During egg deposition which is always accompanied by a moult process, eggs are laid inside the old cuticle. The average clutch contains about 7 eggs with a minimum of 3 and a maximum of 12. The egg laying process usually takes less than two minutes from the first to the last egg. Egg containing cuticles (780 eggs in total) were collected 24 h after egg deposition and washed several times with Volvic™ water. Eggs were not separated from the cuticles because this process would damage the eggs. All samples were frozen in liquid nitrogen and stored at −80°C.

### Sample Preparation and One Dimensional Gel Electrophoresis

The animals (200 individuals each for active and tun state) and eggs (blastula, 780 eggs) were homogenized as described before [15] with the slight modification of adding phosphatase inhibitors to the lysis buffer. Briefly, collected animals/eggs were homogenized in 60 µl lysis buffer containing 8 M urea, 4% CHAPS, 30 mM Tris, Protease Inhibitor Mix (GE Healthcare, München, Germany), Phosphatase Inhibitor Cocktail 1+2 (Sigma-Aldrich, München, Germany) and orthovanadate (50 mM), pH 8.5 by ultrasonication (SONOPULS, HD3100, Bandelin Electronic, Berlin, Germany) with 45% amplitude intensity and 1–0.5 sec intervals at 4°C. Orthovanadate (50 mM) was prepared as described by Thingholm et al. [60]. 20 µl of each Phosphatase Inhibitor Cocktail 1+2 and orthovanadate (50 mM) were added to 1 ml lysis buffer to inhibit phosphatase activity. After homogenization the samples were shock frozen and stored at −80°C. For gel electrophoresis insoluble particles were removed by centrifugation for 2 min at 14,000g and 4°C and the supernatant was quantified using BCA mini-assay. One dimensional gel electrophoresis was performed using precast 4–12% Bis-Tris mini gels (Invitrogen, Karlsruhe, Germany) in MES buffer system. Gels were loaded with 40 µg of protein per lane and stained using protein staining solution from Fermentas (St. Leon-Rot, Germany). The entire lane was cut into 27 equal slices (except slice 26 and 27, which were twice as large) and used for in-gel digestion with trypsin. Since the amount of material is highly limited no biological replicates could be performed.

### Preparation of Peptides and Protein Identification

Tryptic digestion of proteins and extraction of peptides were performed as described [61]. After extraction the solutions were dried in a speed-vac at 37°C for 2 h. Peptides were redissolved in 5 µl 0.1% TFA by sonication for 15 min and were applied for separation using a nanoAcquity UPLC (Waters GmbH, Eschborn, Germany). Peptides were trapped on a nanoAcquity C18 column, 180 µm × 20 mm, particle size 5 µm (Waters GmbH, Eschborn, Germany). The liquid chromatography separation was performed at a flow rate of 400 nl/min on a BEH 130 C18 column, 100 µm × 100 mm, particle size 1.7 µm (Waters GmbH, Eschborn, Germany). Slices 1–22 were analyzed using a 2 h gradient and for slices 23–27 a 1 h gradient was applied. The 2 h gradient was set as follows: from 0 to 4% B in 1 min, from 4 to 30% B in 80 min, from 30 to 45% B in 10 min, from 45 to 90% B in 10 min, 10 min at 90% B, from 90 to 0% B in 0.1 min, and 10 min at 0% B. The 1 h gradient was set as follows: from 0 to 4% B in 1 min, from 4 to 40% B in 40 min, from 40 to 60% B in 5 min, from 60 to 85% B in 0.1 min, 6 min at 85% B, from 85 to 0% B in 0.1 min, and 9 min at 0% B. Solvent A contains 98.9% water, 1% acetonitrile, 0.1% formic acid, solvent B contains 99.9% acetonitrile and 0.1% µl formic acid. The nanoUPLC system was coupled online to an LTQ Orbitrap XL mass spectrometer (Thermo Fisher Scientific, Bremen, Germany). Data were acquired by scan cycles of one FTMS scan with a resolution of 60000 at 400 m/z and a range from 370 to 2000 m/z in parallel with six MS/MS scans in the ion trap of the most abundant precursor ions.

The mgf-files were used for database searches with the MASCOT search engine (Matrix Science, London, UK; version 2.2) against a newly developed tardigrade database containing contigs from 454 sequencing (unpublished data). The peptide mass tolerance for database searches was set to 5 ppm and fragment mass tolerance to 0.6 Da. Carbamidomethylation of C was set as fixed modification. Variable modifications included oxidation of M and deamidation of NQ. In a separate search we selected phosphorylation of S, T and Y as additional modification for the identification of phosphopeptides. One missed cleavage site in case of incomplete trypsin hydrolysis was allowed. Furthermore, proteins were considered as identified if more than one unique peptide had an individual ion score exceeding the MASCOT identity threshold (ion score cut-off of 24). Identification under the applied search parameters refers to a match probability of p<0.01, where p is the probability that the observed match is a random event.

The abundance of proteins was estimated by comparing the exponentially modified Protein Abundance Index (emPAI) [17] which was automatically calculated by the MASCOT search engine. We analyzed each slice separately and avoided to merge the MS/MS data prior to protein database search to maintain the information about molecular weight of each protein. Since emPAI is defined to represent the absolute protein amount we manually calculated the sum of emPAI for proteins that were found repeatedly in different slices. The Protein Abundance Index (PAI) is defined as the number of identified peptides divided by the number of theoretically observable tryptic peptides for each protein, and was later converted to exponentially modified PAI (emPAI, the exponential form of PAI minus one) [17]. The success of using emPAI was demonstrated by determining absolute abundance of 46 proteins in a mouse whole-cell lysate, which had been measured using synthetic peptides [62]. The emPAI can be directly used for reporting approximate protein abundance in a large-scale analysis as shown in different studies [63], [64], [65], [66], [67].

### Preparation of Tardigrade Protein Database

#### Assembly of the 454 sequences

1 million reads from the 454 sequencing and their de novo assembly by Newbler (454/Roche) were received by GATC (http://www.gatc-biotech.com/de/index.html). From the reads 400890 clusters were included in the assembly with 85% aligned reads. The assembly yielded 28345 contigs, 13076 contigs with a length larger than 500 bases.

#### Prediction of the proteins from the EST sequences

FrameDP peptide detection [68] (version 1.0.3; standard parameters) was performed locally on a 2.4 Ghz quad-core desktop computer with 4 Gb RAM running GNU/Linux (Ubuntu 8.10). The learning set was split using the GC3%-method (i.e. GC content of the third codon position) and FrameDP was trained on *M. tardigradums* coding style against *Drosophila melanogaster* protein data as a reference database (Flybase:dmel-all-translation-r5.21.fasta,ftp://ftp.flybase.net/genomes/Drosophila_melanogaster). The annotation of the predicted proteins was performed using BlastX search [69] against Uniprot/Swissprot (version 57.7, September 2009), Uniprot/TrEMBL (version 40.7, September 2009, The UniProt Consortium, 2008) and NRDB (version September, 1^st^ 2009) with an E-value cut-off of 1e-3 and a hmmer2-search against PFAM database (release 23, [70]) with an E-value cut-off of 1e-3.

### Classification of Proteins

For functional analysis of identified proteins we used Blast2GO program, which consists of three main steps: blast to find homologous sequences, mapping to collect GO-terms associated with blast hits and annotation to assign functional terms to query sequences from the pool of GO terms collected in the mapping step [71]. Functional assignment is based on GO database. Sequence data of identified proteins were uploaded as a multiple FASTA file to the Blast2GO software. We performed the blast step against the public NCBInr database using blastp. Other parameters were kept at default values: e-value threshold of 1e-3 and a recovery of 20 hits per sequence. Furthermore, minimal alignment length (hsp filter) was set to 33 to avoid hits with matching regions smaller than 100 nucleotides. QBlast-NCBI was set as Blast mode. An annotation configuration with an e-value-hit-filter of 1.0E-6, Annotation CutOff of 55 and GO weight of 5 have been selected. To grouping all identified proteins in selected subgroups of GO categories (molecular function and biological process) we used the analysis tool of combined graph. To obtain a compact representation of the information, we selected a sequence filter of 20 [72]. The sequence information of proteins in every GO subgroup can be exported as a text file.

### Protein Domain Analysis of Proteins without Annotation

Six frame translations of the author constructed cDNA clusters were run through the DomainSweep pipeline [73] and the significant and putative hits were collected. For each of the protein/domain databases used, different thresholds and rules were established [73]. Domain hits are listed as ‘significant’.

- if two or more hits belong to the same INTERPRO [74] family. The task compares all true positive hits of the different protein family databases grouping together those hits, which are members of the same INTERPRO family/domain.
- if the motif shows the same order as described in PRINTS [75] or BLOCKS [76]. Both databases characterize a protein family with a group of highly conserved motifs/segments in a well-defined order. The task compares the order of the identified true positive hits with the order described in the corresponding PRINTS or BLOCKS entry. Only hits in correct order are accepted.

All other hits above the trusted thresholds are listed as ‘putative’. By comparing the peptides which were identified by mass spectrometry with the six translations, the correct frame and the associated domain information was listed.

## Supporting Information

Table S1 **Identified proteins in early embryonic state (EES).**
(XLSX)

Table S2 **Identified proteins of adults in active state (AS).**
(XLSX)

Table S3 **Identified proteins of adults in tun state (TS).**
(XLSX)

Table S4 **Summary of all identified proteins categorized in protein families and functional groups defined by Gene Ontology and DomainSweep analysis.**
(XLSX)

Table S5 **Summary of proteins phosphorylated at serine and threonine residues.**
(XLSX)

Table S6 **Comparison of major components in active state (AS) determined by emPAI and 2D proteomics data.**
(XLSX)
